# *LncRNA RP5-998N21.4* promotes immune defense through upregulation of IFIT2 and IFIT3 in schizophrenia

**DOI:** 10.1038/s41537-021-00195-8

**Published:** 2022-03-01

**Authors:** Bo Guo, Tingyun Jiang, Fengchun Wu, Hongyu Ni, Junping Ye, Xiaohui Wu, Chaoying Ni, Meijun Jiang, Linyan Ye, Zhongwei Li, Xianzhen Zheng, Shufen Li, Qiong Yang, Zhongju Wang, Xingbing Huang, Cunyou Zhao

**Affiliations:** 1 Department of Medical Genetics, School of Basic Medical Sciences, and Guangdong Technology and Engineering Research Center for Molecular Diagnostics of Human Genetic Diseases, Southern Medical University, Guangzhou, Guangdong China; 2 Key Laboratory of Mental Health of the Ministry of Education, Guangdong-Hong Kong-Macao Greater Bay Area Center for Brain Science and Brain-Inspired Intelligence, and Guangdong Province Key Laboratory of Psychiatric Disorders, Southern Medical University, Guangzhou, Guangdong China; 3 The Third People’s Hospital of Zhongshan, Zhongshan, Guangdong China; 4 Department of Psychiatry, the Affiliated Brain Hospital of Guangzhou Medical University (Guangzhou Huiai Hospital), Guangzhou, Guangdong China; 5 Guangdong Mental Health Center, Guangdong Provincial People’s Hospital, Guangdong Academy of Medical Sciences, Guangzhou, China; 6 Experimental Education/Administration Center, School of Basic Medical Science, Southern Medical University, Guangzhou, China; 7 Department of Rehabilitation, Zhujiang Hospital of Southern Medical University, Guangzhou, China

**Keywords:** Schizophrenia, Epigenetics in the nervous system

## Abstract

Schizophrenia is a complex polygenic disease that is affected by genetic, developmental, and environmental factors. Accumulating evidence indicates that environmental factors such as maternal infection and excessive prenatal neuroinflammation may contribute to the onset of schizophrenia by affecting epigenetic modification. We recently identified a schizophrenia-associated upregulated long noncoding RNA (lncRNA) *RP5-998N21.4* by transcriptomic analysis of monozygotic twins discordant for schizophrenia. Importantly, we found that genes coexpressed with *RP5-998N21.4* were enriched in immune defense-related biological processes in twin subjects and in *RP5-998N21.4*-overexpressing (OE) SK-N-SH cell lines. We then identified two genes encoding an interferon-induced protein with tetratricopeptide repeat (IFIT) 2 and 3, which play an important role in immune defense, as potential targets of *RP5-998N21.4* by integrative analysis of *RP5-998N21.4*_OE_-induced differentially expressed genes (DEGs) in SK-N-SH cells and *RP5-998N21.4*-coexpressed schizophrenia-associated DEGs from twin subjects. We further demonstrated that *RP5-998N21.4* positively regulates the transcription of *IFIT2* and *IFIT3* by binding to their promoter regions and affecting their histone modifications. In addition, as a general nuclear coactivator, RMB14 (encoding RNA binding motif protein 14) was identified to facilitate the regulatory role of *RP5-998N21.4* in *IFIT2* and *IFIT3* transcription. Finally, we observed that *RP5-998N21.4*_*OE*_ can enhance IFIT2- and IFIT3-mediated immune defense responses through activation of signal transducer and activator of transcription 1 (STAT1) signaling pathway in U251 astrocytoma cells under treatment with the viral mimetic polyinosinic: polycytidylic acid (poly I:C). Taken together, our findings suggest that lncRNA *RP5-998N21.4* is a critical regulator of immune defense, providing etiological and therapeutic implications for schizophrenia.

## Introduction

Schizophrenia is a complex genetic disease that affects approximately 1% of the global population^1^. The complexity of schizophrenia is recognized to result from interactions between the genome and the environment^2^. Epidemiological studies have suggested that prenatal exposure to bacterial or viral infection is an important environmental risk factor for schizophrenia^3^. Accumulating evidence suggests that epigenetic modifications may be particularly vulnerable to environmental influences, especially during embryonic development, and play key roles in mediating the interplay between genomic and environmental factors underlying the development of schizophrenia^4^. As one of the major epigenetic modifications, long noncoding RNAs (lncRNAs), which are >200 nt in length, play important roles in the regulation of the immune response through different mechanisms, including acting as signals, decoys, guides, or scaffolds^5^. In recent years, several lncRNAs involved in the inflammatory response or viral infection by binding a target protein have been described^6,7^. For instance, the lncRNA *IVRPIE* promotes the host antiviral immune response by regulating interferon (IFN) β1 and IFN-stimulated gene (ISG) expression^8^. The secretion of IFNs from host cells is a response to various pathogens, such as viruses, bacteria, fungi, or parasites, and induces a protective immune defense. IFNs exert their antiviral effects via Janus kinase (JAK)/STAT-mediated signaling through IFN receptors, leading to the induction of approximately 300 ISGs. Several ISG proteins are structurally characterized by tetratricopeptide repeats, and they are called IFN-induced proteins with tetratricopeptide repeats (IFITs). IFIT2 and IFIT3 are IFITs; IFIT3 (ISG60) is also involved in poly I:C-induced CXCL10 expression through the Toll-like receptor 3 (TLR3)/IFN-β/STAT1 axis in U373MG human astrocytoma cells^9^, suggesting that IFITs may play important roles in a variety of biological processes, including physiological innate immunity and pathological inflammation in the central nervous system.

We previously performed transcriptomic analysis of monozygotic twins discordant for schizophrenia and identified two schizophrenia-associated upregulated lncRNAs, *AC006129.1* and *RP5-998N21.4*, whose coexpressed genes are involved in immune and defense response-related biological processes^10^. We delineated the mechanism by which *AC006129.1* binds to the promoter region of the transcriptional repressor Capicua (CIC) and promotes DNA methylation-mediated CIC downregulation by facilitating the interactions of DNA methyltransferases with the *CIC* promoter, thereby alleviating CIC-induced suppressor of cytokine signaling 3 (SOCS3) repression. Derepression of SOCS3 enhances the anti-inflammatory response by inhibiting JAK/STAT-signaling activation. However, the underlying gene regulatory mechanism that mediates the roles of another disease-associated lncRNA *RP5-998N21.4* in the context of schizophrenia remains unclear.

In this study, we performed integrative RNA sequencing data analysis of monozygotic twin discordant for schizophrenia, a *lncRNA RP5-998N21.4* overexpressing SK–N–SH cell line, and public postmortem brain datasets to identify the potential coexpressed target genes of *RP5-998N21.4*, and we then identified the mechanism by which *RP5-998N21.4* underlies the development of schizophrenia by regulating IFIT2- and IFIT3-mediated antiviral immune response pathways.

## Results

### Upregulation of *RP5-998N21.4* in schizophrenia is involved in immune response-related pathways

**Fig. 1:**
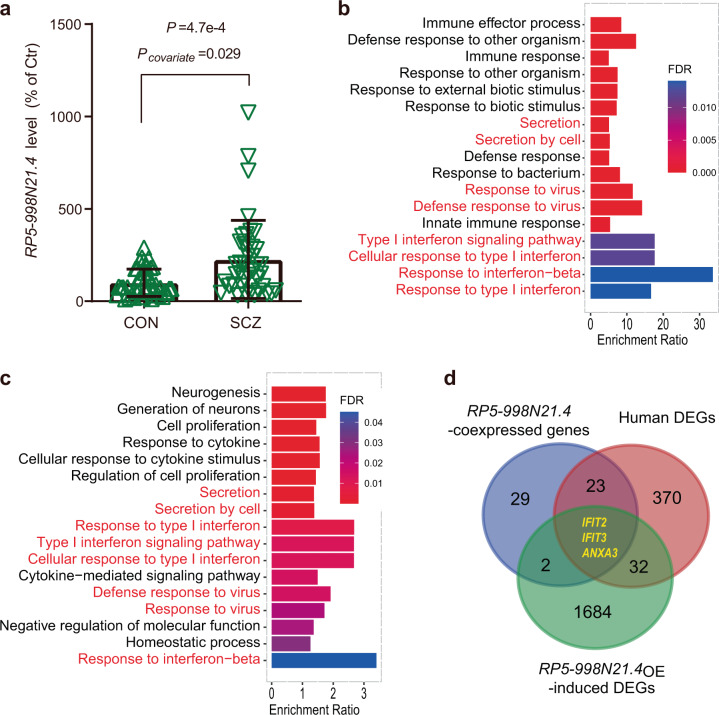
Upregulation of *RP5-998N21.4* in schizophrenia is involved in immune defense-related pathways. **a** The *RP5-998N21.4* level in peripheral blood samples from the validation cohort, namely, 51 patients with schizophrenia (SCZ) and 48 nonpsychiatric controls (CON). The expression levels in SCZ and CON are shown as percentages relative to the mean level in CON with standard deviations (s.d.) indicated by error bars. Significant differences between the two cohorts were determined with ANCOVA without covariates (*P* < 0.05) or with age and sex as covariates (*P*covariate). **b**, **c** Functional enrichments of GO–BP annotations among *RP5-998N21.4*-coexpressed genes in human twin subjects (**b**) or among *RP5-998N21.4*_*OE*_-induced mouse DEGs (**c**) are shown for the top gene sets. **d** Venn diagram showing overlapping relationships. The numbers indicate the gene counts among the *RP5-998N21.4*-coexpressed genes in humans, the schizophrenia-associated DEGs (human DEGs) from twin subjects, and the *RP5-998N21.4*_*OE*_ DEGs from SK–N–SH cells. All blots were derived from the same experiments and were processed in parallel.

We previously found that the lncRNA *RP5-998N21.4* (also called ENSG00000234571, *NONHSAT225469.1*, or *NONHSAG105013.1*) was upregulated in the patient within monozygotic twins discordant for schizophrenia (Log_2_FC = 1.58 and FDR = 0.006)^10^. We then validated its expression level in blood samples of an independent validation cohort containing 51 schizophrenia patients and 48 nonpsychiatric controls. qPCR analysis revealed significantly increased *RP5-998N21.4* expression levels in patients with schizophrenia compared with nonpsychiatric controls (increased by 226%, *P* < 0.001 and *P*_covariate_ = 0.029 when age and sex were included as covariates in ANCOVA; Fig. 1a). Moreover, upregulation of *RP5-998N21.4* in blood samples of patients with schizophrenia remained significant in the CommonMind Consortium (CMC) brain RNA-seq dataset (Log_2_FC = 0.03, *P* = 0.01)^11^ and met the trend line in the PsychENCODE (Log_2_FC = 0.11, *P* = 0.17)^12^ and Children’s Hospital of Philadelphia (CHP, Log2FC = 0.20, *P* = 0.48)^13^ brain RNA-seq datasets. To explore the biological implications of lncRNA-*RP5-998N21.4* in schizophrenia, we identified 367 mRNAs correlated with lncRNA-*RP5-998N21.4* expression (|coefficient *r*| > 0.5, *P* < 0.05) among the 16 individuals from the same four pairs of schizophrenia-discordant twins (SDC) and four pairs of healthy concordant control twins (HCC) included in the above lncRNA analysis (Supplementary Table 1). We then used a weighted gene coexpression network analysis (WGCNA) approach to perform coexpression analyses of the lncRNA *RP5-998N21.4* and these 367 mRNAs in these sixteen individuals and identified that 57 mRNAs were coexpressed with lncRNA *RP5-998N21.4* in the brown module (Supplementary Fig. 1 and Supplementary Table 2) and were also significantly enriched in whole blood, minor salivary gland, and spleen tissues based on the GTEx RNA-seq dataset (Supplementary Table 3)^14^. Gene ontology–biological pathway (GO–BP) analysis revealed significant enrichment of the 57 coexpressed genes in terms such as “immune response”, “immune effector process”, “defense response to virus”, “type I IFN signaling pathway”, and “response to IFN-beta” (Fig. 1b and Supplementary Data 1), suggesting functional implications of *RP5-998N21.4* in the pathogenesis of schizophrenia.

As annotated in the University of California, Santa Cruz (UCSC) database, the *RP5-998N21.4* transcript is located on the negative strand of chromosome 1 (chr1:149320440–149379646, hg19) and exists in one isoform of 3540 nt with two exons (Supplementary Fig. 2). The in silico results obtained with the open reading frame (ORF) prediction tool ORFfinder and the Phylogenetic Codon Substitution Frequency tool to distinguish coding and noncoding transcripts consistently showed that *RP5-998N21.4* has no potential protein-coding ability (Supplementary Fig. 3). We also observed that *RP5-998N21.4* was highly expressed in human white blood cells and the SK–N–SH cell line, as determined by qPCR, and was moderately expressed in whole blood and brain tissues from published NONCODE and GTEx RNA-seq datasets (Supplementary Fig. 4a–c)^14,15^. We then characterized the subcellular localization of *RP5-998N21.4* and observed that it was expressed predominantly in the nucleus (Supplementary Fig. 4d), as determined by qPCR in HEK293T cells and RNA-seq data in SK–N–SH cells from the lncATLAS dataset^16^.

To further elucidate the molecular mechanism underlying the upregulation of *RP5-998N21.4*, we overexpressed a recombinant lentiviral vector containing *RP5-998N21.4* in SK–N–SH cells. RNA-seq analysis identified 1722 differentially expressed genes (DEGs, DE-seq: |LogFC| > 0.5, FDR < 0.01) (Supplementary Data 2) induced by *RP5-998N21.4*_OE_. GO–BP analysis revealed significant enrichment of the 1722 DEGs in terms such as “neurogenesis”, “type I IFN signaling pathway”, “defense response to virus”, and “response to IFN-beta” (Fig. 1c and Supplementary Data 3), further supporting the role of *RP5-998N21.4* related to immune response pathways. We then observed significant overlap (three overlapping genes (*IFIT2*, *IFIT3*, and *ANXA3;* OR = 77.4, *P* = 7.4e−5; Fig. 1d) between *RP5-998N21.4*_*OE*_-induced DEGs in SK-N-SH cells and *RP5-998N21.4-*coexpressed schizophrenia-associated DEGs in twin subjects. These three genes were upregulated consistently in *RP5-998N21.4*_OE_ SK cells and in MZ twin patients, and their expression was positively correlated with *RP5-998N21.4* expression. Moreover, coexpression patterns of *RP5-998N21.4* with *IFIT2*, *IFIT3*, and *ANXA3* were further observed in whole blood and brain tissue from the GTEx RNA-seq dataset (Supplementary Data 4)^14^. IFIT2 and IFIT3 have been reported to positively regulate TLR3/IFN-β/phosphorylation of the STAT1 axis in U373MG human astrocytoma cells^9^ and play important roles in the antiviral response^17,18^. The upregulation of *IFIT2* and *IFIT3* observed in our twin patients was also observed to be significant in a lymphoblastoid cell lines (LCL) study (*IFIT2:* *β* = 0.4, FDR = 1.7e−12; *IFIT3:* *β* = 0.5, FDR = 7.8e−15)^19^ and to meet the trend line in the CMC brain study (*IFIT2:* Log2FC = 0.09 and *P* = 0.05; *IFIT3:* Log2FC = 0.02, *P* = 0.7)^11^. *ANXA3*, as a marker of brain microglia^20^, is reported to be associated with cell death in lactacystin-mediated neuronal injury^21^ and to inhibit the PI3K/Akt signaling pathway^22,23^. The upregulation of *ANXA3* observed in our twin patients was also supported by the LCL study (*β* = 0.2, FDR = 1.4e−3)^19^ and a PsychENCODE brain dataset (LogFC = 0.08 and FDR = 0.07)^12^. Collectively, these results indicate that *RP5-998N21.4* might regulate immune defense-related pathways by promoting the transcription of *IFIT2*, *IFIT3*, or *ANXA3*, which are involved in the pathophysiology of schizophrenia.

### *RP5-998N21.4* promotes IFIT3 and IFIT2 transcription by affecting chromatin modifications

**Fig. 2:**
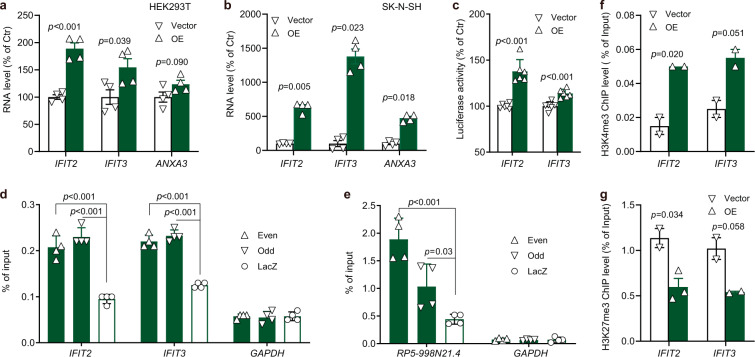
*RP5-998N21.4* regulates its targets through histone modifications. **a**–**c** Effects of *RP5-998N21.4*_OE_ on the endogenous expression of potential target genes in HEK293T cells (**a**) and in SK-N-SH cells in panel (**b**) or on *IFIT2* and *IFIT3* promoter activities in luciferase reporter assays in HEK293T cells (**c**). **d**, **e** ChIRP assay of the interaction of *IFIT2* or *IFIT3* with *RP5-998N21.4* in *RP5-998N21.4*_*OE*_ HEK293T cells. The retrieved *IFIT2* and *IFIT3* DNA (**d**) or lncRNA-*RP5-998N21.4* (**e**) from the lncRNA *RP5-998N21.4* ChIRP assay was quantified by qPCR, and the data in columns are expressed as the percentage of input. Data for the even (Δ: #2, 4, 6)- and odd (∇: #1, 3, 5)-numbered probes of *RP5-998N21.4*, as well as the *LacZ* probe (О) as the negative control, are shown in the panels. **f**, **g** ChIP-qPCR of H3K4me3 (**f**) and H3K27me3 (**g**) enrichment at the promoter regions of *IFIT2* and *IFIT3* in HEK293T cells with or without *RP5-998N21.4*_OE_. The white column represents the blank control group, and the green column represents the *RP5-998N21.4*_*OE*_ group. The *p* value from Student’s *t* test is shown between the indicated comparisons. The data are shown with Δ or ∇ and are presented as the mean ± s.d. values. All blots were derived from the same experiments and were processed in parallel.

We further explored the mechanism involved in *RP5-998N21.4*-mediated regulation of *IFIT2*, *IFIT3*, and *ANXA3* expression. It has been reported that lncRNAs can act as scaffolds by interacting with protein partners to facilitate their binding to target genes to induce their transcription^7^. We first confirmed that *RP5-998N21.4* overexpression (*RP5-998N21.4*_OE_) increased *IFIT2* and *IFIT3* expression levels in HEK293T and SK–N–SH cells and increased the *ANXA3* expression level only in SK–N–SH cells (Fig. 2a, b). We then used LongTarget to predict several binding sites of *RP5-998N21.4* in the proximal promoter regions of *IFIT2* and *IFIT3* and validated the upregulation of *IFIT2* and *IFIT3* expression by *RP5-998N21.4*_OE_ in HEK293T cells cotransfected with pcDNA-*RP5-998N21.4* and a luciferase reporter vector driven by the *IFIT2* or *IFIT3* promoter (Fig. 2c).

Next, we tested the binding activities of *RP5-998N21.4* to the *IFIT2* or *IFIT3* promoter region using chromatin isolation by RNA purification (ChIRP) assay and observed that *RP5-998N21.4* can bind to the proximal promoter regions of *IFIT2* and *IFIT3*, as determined by qPCR analysis of the retrieved DNA (Fig. 2d) or RNA (Fig. 2e) from *RP5-998N21.4*-ChIRP. Finally, we examined whether the binding of *RP5-998N21.4* to the *IFIT2* or *IFIT3* promoter region affects the chromatin state by performing chromatin immunoprecipitation (ChIP)-qPCR for a transcriptionally active histone mark (H3K4me3) and a repressive histone mark (H3K27me3). Notably, H3K4me3 enrichment at the *IFIT2* promoter region was significantly higher in *RP5-998N21.4*_OE_ HEK293T cells than in control cells (Fig. 2f). In contrast, H3K27me3 enrichment at the *IFIT2* promoter region was significantly lower in *RP5-998N21.4*_OE_ cells than in control cells (Fig. 2g). Similarly, marginal alterations in enrichment were also observed for H3K4me3 (*p* = 0.051; Fig. 2f) and H3K27me3 (*p* = 0.058; Fig. 2g) at the *IFIT3* promoter region. However, we did not detect significant H3K4me3 and H3K27me3 enrichment at the *ANXA3* transcriptional start site region in *RP5-998N21.4*_OE_ cells and the corresponding control cells (Supplementary Fig. 5), indicating that there may be other mechanisms involved in *ANXA3* transcription. In summary, these results indicate that *RP5-998N21.4* regulates the transcription of *IFIT2* and *IFIT3* by binding to their promoter regions and affecting histone modifications in these promoter regions.

### RBM14 facilitates the regulatory role of *RP5-998N21.4* in IFIT2 and IFIT3 transcription

**Fig. 3:**
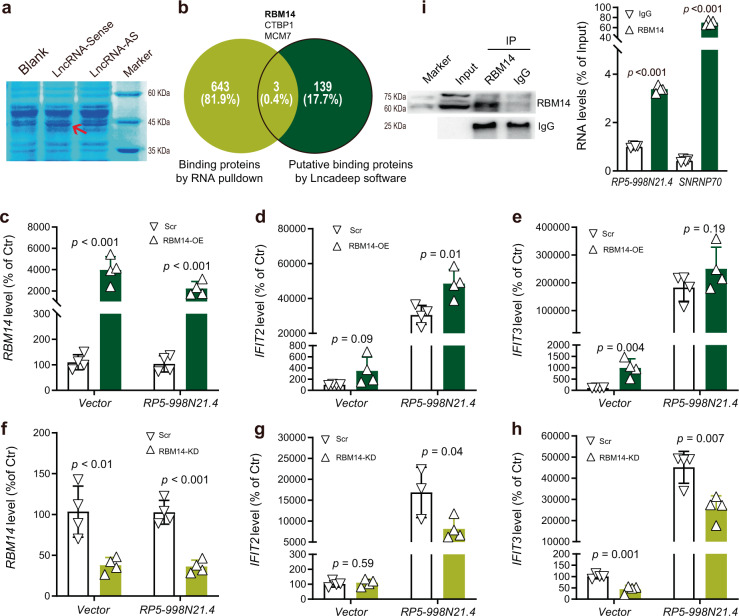
Effects of the protein interacting partner RBM14 on the regulatory role of *RP5-998N21.4* in HEK293T cell. **a** Coomassie Brilliant Blue staining of the proteins pulled down by *PR5-998N21.4* from HEK293T cell lysates; the band indicated by the arrow was sent for mass spectrometry analysis. **b** Venn diagram of the 646 interacting proteins predicted by LncADeep and the 142 binding proteins identified by mass spectrometry. **c**–**e** Effects of *RBM14* overexpression (**c**) on the regulatory role of *RP5-998N21.4* in *IFIT2* (**d**) and *IFIT3* (**e**) transcription. **f**–**h** Effects of *RBM14* knockdown (**f**) on the regulatory role of *RP5-998N21.4* in *IFIT2* (**g**) and *IFIT3* (**h**) transcription. **i** RNA immunoprecipitation (RIP) combined with qPCR was performed to detect the association of RBM14 with lncRNA-*RP5-998N21.4* in HEK293T cells. The immunoblots show the products of immunoprecipitation (IP, left) with the anti-RMB14 antibody, and the qPCR analysis results (right) show that *RP5-998N21.4* was abundant in the RBM14 immunoprecipitates of HEK293T cells. Immunoglobin G (IgG) served as the negative immunoprecipitation control, and SNRNP70 served as the positive control for interaction with RBM14. The *p* value from the two-tailed Student’s *t* test is shown between the indicated two groups. The data from each group are shown with Δ or ∇ and are presented as the mean ± s.d. values. All blots were derived from the same experiments and were processed in parallel.

We next explored the associated proteins that interact with *RP5-998N21* using *in silico* prediction analysis and an in vivo experiment. We first employed LncADeep software to identify 142 proteins that were predicted to significantly interact with *RP5-998N21.4* (score > 0.5, Supplementary Data 5)^24^. We then performed an RNA pulldown assay using in vitro-transcribed biotinylated *RP5-998N21.4* or its antisense control RNA to pull down protein partners from nuclear extracts of HEK293T cells. RNA–protein complexes were captured with streptavidin magnetic beads, separated by sodium dodecyl sulfate-polyacrylamide gel electrophoresis (SDS-PAGE), and stained with coomassie brilliant blue, and the bands of interest were excised and sent for mass spectrometry analysis (Fig. 3a and Supplementary Fig. 6a). This approach identified 646 proteins that bound significantly to *RP5-998N21.4* (peptide expectation value < 0.05, Supplementary Data 6), and three of the 646 proteins RBM14, CTBP1, and MCM7 were also predicted to bind to *RP5-998N21.4* by the above mentioned LncADeep software (Fig. 3b).

We further found that overexpression of *RBM14* (Fig. 3c) significantly increased the mRNA level of endogenous *IFIT2* (Fig. 3d) and marginally increased that of *IFIT3* (Fig. 3e) in HEK293T cells with or without cotransfection of *RP5-998N21.4*, whereas overexpression of *MCM7* and *CTBP1* did not increase the endogenous *IFIT2* and *IFIT3* mRNA levels in *RP5-998N21.4*_OE_ HEK293T cells (Supplementary Fig. 6b, c). We also employed shRNA to achieve knockdown (KD) of endogenous *RBM14* expression (Fig. 3f) and observed that *RBM14-*KD significantly attenuated the increases in *IFIT2* (Fig. 3g) and *IFIT3* (Fig. 3h) transcription induced by *RP5-998N21.4*_OE_ in HEK293T cells. Moreover, RNA immunoprecipitation (RIP) assay using an anti-RBM14 antibody followed by qPCR showed that *RP5-998N21.4* was apparently present in the immunoprecipitates of RBM14 from HEK293 cells (Fig. 3i and Supplementary Fig. 7). RBM14 (RNA binding motif protein 14) encodes a ribonucleoprotein that functions as a general nuclear coactivator and an RNA splicing modulator^25^. *RBM14* has been identified to be significantly upregulated in postmortem brain tissues in the PsychENCODE database (Log2FC = 0.072, FDR = 0.001)^12^ and marginally upregulated in the LCL dataset (*β* = 0.122, FDR = 0.098)^19^ of patients with schizophrenia compared to nonpsychiatric controls in public RNA-seq datasets. These observations suggest that RBM14 facilitates the regulatory role of *RP5-998N21.4* in *IFIT2* and *IFIT3* transcription.

### *RP5-998N21.4* promotes the immune response by activating STAT1 signaling

**Fig. 4:**
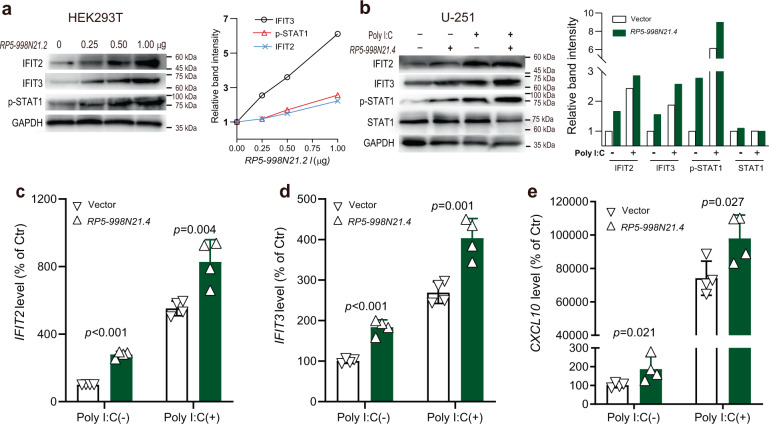
*RP5-998N21.4* promotes immune responses through activation of STAT1 signaling. **a** Activation of STAT1 signaling was enhanced by *RP5-998N21.4*-induced dose-dependent upregulation of IFIT2 and IFIT3 in HEK293T cells. Protein extracts from HEK293T cells with *RP5-998N21.4*_OE_ were immunoblotted with the indicated antibodies, as shown in the left panel, and the band intensities relative to that of the vector control are shown in the right panel. **b**–**e** *RP5-998N21.4* enhanced poly I:C-induced upregulation of IFIT2 and IFIT3 and activation of STAT1 signaling in U251 human astrocytoma cells, as determined by western blot (**b**) and qPCR analyses of *IFIT2*, *IFIT3*, and *CXCL10* RNA levels (**c**–**e**). U251 cells were stimulated with poly I:C (0, 30 μg/ml) for another 4 h and were then collected for immunoblot analysis 44 h after transfection with or without *RP5-998N21.4*. Protein extracts from cells transfected with or without *RP5-998N21.4* were immunoblotted with the indicated antibodies in the left panel in (**b**), and the band intensities are shown in the right panel in (**b**). The *p* value from the two-tailed Student’s *t* test is shown between the indicated two groups. The data from each group are shown with ∇ (vector control) or Δ (*RP5-998N21.4*_*OE*_) and are presented as the mean ± s.d. values. All blots were derived from the same experiments and were processed in parallel.

Since IFIT2 and IFIT3 have been reported to play important roles in the antiviral response through the promotion of TLR3/IFN-β/STAT1 axis activity^9^, we then examined whether *RP5-998N21.4*-mediated upregulation of IFIT2 and IFIT3 activates STAT1 signaling. We first observed that activation of STAT1 was enhanced by *RP5-998N21.4*-induced dose-dependent upregulation of IFIT2 and IFIT3 in HEK293T cells (Fig. 4a and Supplementary Fig. 8). We next used the viral mimetic poly I:C to experimentally model viral infection in U251 astrocytoma cells and evaluated the role of *RP5-998N21.4* in the response to infection mediated through upregulation of *IFIT3* expression (Supplementary Fig. 9). We observed that poly I:C significantly induced the protein levels of IFIT2, IFIT3, and phosphorylated STAT1 (Fig. 4b and Supplementary Fig. 10), as well as the RNA levels of *IFIT2*, *IFIT3*, and *CXCL10* (Fig. 4c–e), which were enhanced by *RP5-998N21.4* overexpression in U251 cells. These results indicate that *RP5-998N21.4* elicits an antiviral response through upregulation of IFIT2- and IFIT3-mediated activation of the STAT1 signaling pathway.

## Discussion

The present findings reveal that upregulation of *RP5-998N21.4* promotes the activity of immune response-related pathways through upregulation of IFIT2 and IFIT3 in the context of schizophrenia and that overexpression of this lncRNA can enhance IFIT2- and IFIT3-mediated antiviral defense responses through activation of STAT1 signaling pathways in human U251 astrocytoma cells treated with the viral mimetic poly I:C. This epigenetic mechanism that links lncRNAs to the promotion of immune defense responses has promising therapeutic implications for schizophrenia.

Schizophrenia is a multifactorial neurodevelopmental disorder with genetic and environmental etiologies. Several studies indicate that prenatal viral/bacterial infections, inflammation, and immune activation increase the offspring’s risk for developing schizophrenia.^26^ Recently, lncRNAs have emerged as potential key regulators of inflammatory responses^7^. In our previous study, transcriptomic analysis of monozygotic twins discordant for schizophrenia identified two schizophrenia-associated upregulated lncRNAs, *AC006129.1* and *RP5-998N21.4*; *AC006129.1* was identified to reactivate the SOCS3-mediated anti-inflammatory response through DNA methylation-mediated CIC downregulation in schizophrenia^10^. In this study, we further confirmed that schizophrenia-associated upregulation of *RP5-998N21.4* in the patient within SDC twins remained significant in blood samples from an independent sporadic cohort as well as in a previously published CMC postmortem brain RNA-seq dataset^11^. Moreover, we demonstrated that *RP5-998N21.4*_OE_-induced coexpressed DEGs in SK–N–SH cell lines were significantly enriched in immune defense-related pathways; two antiviral response-related genes, *IFIT2* and *IFIT3*, were significantly enriched in both *RP5-998N21.4-*coexpressed schizophrenia-associated DEGs and *RP5-998N21.4*_OE_-induced DEGs and were identified as potential targets of lncRNA *RP5-998N21.4*. More importantly, consistent with the upregulation of *IFIT2* and *IFIT3* expression observed in the schizophrenia patients of MZ twin pairs, their expression levels also showed significant alterations in a large sample LCL study^27^, but not in the CMC and PsychENCODE brain studies^11^. Collectively, these results indicate the important role of *RP5-998N21.4* in regulating immune response-related pathways involved in the pathogenesis of schizophrenia.

We identified the epigenetic mechanism by which enhancement of *IFIT2* and *IFIT3* expression by *RP5-998N21.4* promotes antiviral defense response pathway activity through the activation of STAT1 signaling pathways in human cells. We demonstrated that *RP5-998N21.4* can promote the expression of *IFIT2* and *IFIT3* by binding to their proximal promoter regions and then affecting their histone modifications. The interactions between *RP5-998N21.4* and *IFIT2* or *IFIT3* were revealed by a luciferase reporter assay with a vector containing the *RP5-998N21.4-*binding regions and by a ChIRP assay. Binding of *RP5-998N21.4* at the *IFIT2* and *IFIT3* promoter regions altered their histone modifications (the activating mark H3K4me3 and repressive mark H3K27me3), further supporting the regulatory role of *RP5-998N21.4* in activating *IFIT2* and *IFIT3* transcription through chromatin remodeling. We further demonstrated that the regulatory role of *RP5-998N21.4* in *IFIT2* and *IFIT3* transcription was facilitated by RBM14. RBM14 has been identified as a component of nuclear paraspeckles^28^ and shown to modulate the transcription and splicing of host genes in response to viral infection^29^. Intriguingly, upregulated IFIT2 and IFIT3 are also involved in the response to viral infection or immune activation and serve as an essential primary barrier to viral infection^8,18,30^. We further demonstrated that STAT1 signaling was activated by *RP5-998N21.4*-induced upregulation of IFIT2 and IFIT3 in U251 cells under treatment with the viral mimetic poly I:C, in which the *CXCL10* expression level was also upregulated. Recently, IFIT2 and IFIT3 were reported to positively regulate the expression of CXCL10 through activation of the TLR3/IFN-beta/STAT1 axis in U373MG cells treated with poly I:C^9^. CXCL10 is a C–X–C chemokine member that and functions by binding to a specific receptor, CXCR3, and promotes lymphocyte chemotaxis and microglial recruitment. CXCL10 is also reported to be involved in the pathogenesis of Alzheimer’s disease and cerebral ischemia, and astrocyte-derived CXCL10 is reported to suppress oligodendrocyte progenitor cell differentiation^9^. Recent studies have also demonstrated altered cytokine activity in schizophrenia,^31–34^ and enhancement of *IFIT2* and *IFIT3* by *RP5-998N21.4* and the subsequent promotion of the immune defense response might partially counteract the defects caused by the disease, with protective or restorative effects against neurotoxicity. Understanding the biological mechanism underlying the regulatory role of *RP5-998N21.4* in the immune defense response may lead to promising interventions for schizophrenia.

Although our studies demonstrate the involvement of *RP5-998N21.4* in the development of schizophrenia through the enhancement of IFIT2- and/or IFIT3-mediated immune defense response pathways, it is currently unclear how *RP5-998N21.4* influences synapse morphology and function. Furthermore, since most of the function and mechanism study for *RP5-998N21.4* in HEK293T cells, neuronal cells, such as primary neurons or neuron-like cells, may be needed to validate the role of *RP5-998N21.4* in the regulation of immune defense response in the future.

In conclusion, our findings illustrate an epigenetic mechanism by which upregulation of lncRNA *RP5-998N21.4* underlies the development of schizophrenia via the enhancement of IFIT2- and IFIT3-mediated immune defense responses through activation of STAT1 signaling pathways. This epigenetic mechanism that links lncRNAs to the promotion of immune defense responses has promising therapeutic implications for schizophrenia.

## Methods

### Human subject analysis

Peripheral blood samples from four pairs of SDC and four pairs of HCC were employed for strand-specific RNA-seq to identify schizophrenia-associated differentially expressed lncRNAs and mRNAs as described^10^. Briefly, DE-lncRNAs and DE-mRNAs were first identified by edgeR pairwise analysis of four schizophrenia cases versus four healthy controls (four SDC twins) and then retained their significant expression differences in case–control analysis of four schizophrenia cases (from four SDC twins) vs. 12 healthy controls (by including four HCC twins). A validation cohort including 51 patients with schizophrenia and 48 unrelated non-psychotic controls was further employed to measure *RP5-998N21.4* expression level by using qRT-PCR. All patients meet the diagnostic criteria for schizophrenia according to the fourth edition of the Diagnostic and Statistical Manual of Mental Disorders (American Psychiatric Association). Study participants were free of any diagnosis of mental deficiency, traumatic brain injury, or a history of illicit drug abuse or alcoholism. The control group had no present, past or family history of mental illness or substance abuse. The study was approved by the university review from Southern Medical University and the local medical ethics committees of all participating hospitals and universities and is compliant with the ‘Guidance of the Ministry of Science and Technology (MOST) for the Review and Approval of Human Genetic Resources. After introducing the nature of the procedure, the written informed consent of the participants was obtained before the study.

We then used a public RNA-seq dataset to validate the expression differences of interested lncRNA or mRNA between schizophrenia and controls from postmortem brain of CMC dorsolateral prefrontal cortex RNA-seq data^11^ and CHP amygdala RNA-seq data^13,35^ and PsychENCODE RNA-seq dataset (http://www.psychencode.org)^12^, or LCLs of 514 schizophrenia and 690 controls^19,27^.

LncRNA *RP5-998N21.4* co-expressed network analysis was performed with WGCNA in the R package^36^ using FPKM values of lncRNA *RP5-998N21.4* and mRNAs that displayed significant correlations with *RP5-998N21.4* (|coefficient R| > 0.5 and *P* < 0.05) among these sixteen individuals, and network construction and module detection were analyzed with the “BlockwiseModules” function in the WGCNA package. Coexpression patterns of lncRNA *RP5-998N21.4* with the candidate mRNAs were further validated with RNA-seq datasets from brain or blood tissues among at least 40 samples of the GTExv7project (https://dbgap.ncbi.nlm.nih.gov)^14^ by using cor function in R (v3.5.1). GO–BP enrichment analyses were performed using WEB-based GEne SeT AnaLysis Toolkit (WebGestalt: http://www.webgestalt.org/option.php)^37^.

### RNA-seq analysis of *RP5-998N21.4* overexpressed SK–N–SH cells

To explore the potential targets of lncRNA-*RP5-998N21.4*, we overexpressed a recombinant lentivirus carrying lncRNA-*RP5-998N21.4* in SK–N–SH cells and then performed RNA-seq analysis to identify the DE-mRNAs induced by the overexpression of *RP5-998N21.4*. The recombinant lentivirus driven by the EF1a promoter was constructed by cloning the cDNA sequence of *RP5-998N21.4* (Chr1:149372292–149379646, hg19) into the PHAGE-fullEF1a-MCS-IZS Green expression vector via the NhEI and BamHI restriction sites. An empty lentivirus vector with no insert was used as a negative control. To obtain the lentiviruses, the transfer lentiviral plasmid was cotransfected into HEK293T cells with the packaging plasmids pMD2.G and psPAX2 using the CaPO_4_ coprecipitation method as previously described^38^. Transduction of viral particles into SK–N–SH cells was performed according to the manufacturer’s protocol. RNA extracted from the transduced SK–N–SH cells using TRIzol reagent (Invitrogen) with a minimum RNA integrity value of seven was used to build an RNA library. The library was sequenced to a depth of ~27 million 150-bp paired-end reads per sample on an Illumina HiSeq X ten by Novogene Solution (Tianjin, China). The raw reads were subjected to quality control with FastQC, and clean reads generated from the raw reads with Trimmomatic were mapped to the hg19 reference genome using Bowtie2. Differential expression tests were performed using DEseq2 in the R package.

### Cell culturing, plasmid construction, quantitative PCR, Western blotting, and ChIP

Human embryonic kidney (HEK) 293 T cells, human neuroblastoma cells (SK–N–SH), and human U251 astrocytoma cells were cultured at 37 °C and 5% CO_2_ concentrations in DMEM supplemented with 10% fetal bovine serum (EX CELL) and antibiotics (penicillin and streptomycin, Gibco, USA). The U251 cells were stimulated with 30 µg/ml poly I:C (dissolved in PBS, ThermoFisher, USA) for 4 h and collected for further analysis 44 h after transfection with plasmid using Lipofectamine 2000 (Life Technologies, USA).

The cDNAs from *RP5-998N21.4*, *RBM14*, *CMC7*, and *CTBP1* genes were cloned into the pcDNA 3.1(+) plasmid. *RBM14*-shRNAs were synthesized by Sangon Biotech (China) and cloned into a pLKO.1 vector via the AgeI/EcoRI restriction enzyme sites. The *IFIT2* (chr10: 91061628-91061686, hg19) and *IFIT3* (chr10: 91087690–91087750, hg19) proximal promoter sequences were amplified from human genomic DNA and cloned into a pGL4.18 dual-luciferase vector (Promega, USA) via the KpnI/XhoI (for *IFIT2*) or KpnI/HindIII (for *IFIT2*) restriction sites. These reporter constructs were transiently co-transfected into HEK293T cells together with the pRL-TK plasmid as an internal control for transfection efficiency using Lipofectamine 2000 reagents (Invitrogen, USA). Cells were harvested 48 h after transfection, and the dual-luciferase activity (Promega) was measured with the Wallac Victor V 1420 Multilabel Counter (PerkinElmer, USA).

Total RNA extracted from cell lines, human peripheral blood using TRIzol reagent (Life Technologies) was reverse-transcribed into cDNA using a PrimeScript RT Reagent Kit with gDNA Eraser (Takara Japan). A comparative qPCR assay with SYBR green dye-containing SuperArray PCR master mix (YEASEN, China) was performed on an ABI Prism 7900 system (Life Technologies) with *ACTB* and/or *GAPDH* as reference genes for the quantification of target gene levels as 2^−ΔΔCt^ were subjected to statistical analyses. All primers used in this study were synthesized by Sangon Biotech (Supplementary Table 4). Western blotting was performed with rabbit anti-IFIT2 (1:1000, Proteintech, USA), anti-IFIT3 (1: 1000, Proteintech), anti-STAT1 (1: 1000, Proteintech), anti-phosphorate STAT1 (1: 1000, Proteintech), anti-RBM14 (1: 1000, Abclonal, China), rabbit anti-GAPDH (1: 3000, Proteintech) antibodies.

ChIP assays were performed with cell extracts from HEK293T cells using anti-H3K4me3 (1: 100, Abclonal) or anti-H3K27me3 (1: 100, Abclonal) antibodies as recommended (EZ-ChIP, Merck, Germany). Cell lysates extracted from HEK293T cells were subjected to ChIP assays using antibodies 48 h after transfection with pcDNA3.1-RP5-998N21.4 or an empty control vector, and the immunoprecipitated DNA was quantified by qPCR using primers *IFIT3*, *IFIT2*, and *ANXA3* to evaluate the histone modification levels of H3K4me3 and H3K27me3 (Abclonal) at the target regions.

### RNA pulldown assay and mass spectrometry

To explore the protein partners that interact with *RP5-998N21*, we performed in silico predictions and an in vivo experiment. We first employed LncADeep software, which is based on deep learning algorithms^24^, to predict lncRNA-protein interactions and obtain functional annotations for lncRNAs. We then performed an RNA pulldown assay using in vitro-transcribed biotinylated *RP5-998N21.4* or its antisense control RNA to pull down protein partners from HEK293T cell lysates. RNA–protein complexes were captured by streptavidin magnetic beads, separated by SDS-PAGE, and stained with Coomassie Brilliant Blue (Supplementary Fig. 6a). The bands of interest were then excised and sent for mass spectrometry analysis. Briefly, biotin-labeled lncRNAs were transcribed in vitro using a Ribo RNAmax-T7 Biotin Labeling Transcription Kit (RiboBio, China). Biotinylated sense or antisense lncRNA-*RP5-998N21.4* was incubated with HEK293T cell lysates at 4 °C for 2 h. The interacting complexes were then purified with streptavidin agarose beads (S1420, New England Biolabs, USA). The eluted proteins were separated by SDS-PAGE and stained with Coomassie Brilliant Blue, and the interesting protein bands were excised for in-gel trypsin digestion prior to liquid chromatography (LC)–tandem mass spectrometry (MS)/MS analysis at Guangzhou Fitgene Biotechnology Company (China). Proteins with a peptide expectation value <0.05 were considered potential lncRNA-*RP5-998N21.4* interacting partners.

### ChIRP assay

We employed ChIRP to examine the interaction of lncRNA *RP5-998N21.4* with the *IFIT2* and *IFIT3* promoter region using an EZMagna ChIRP RNA Interactome Kit (Merck). Briefly, HEK293T cell lysates were cross-linked with 1% glutaraldehyde 48 h after transfection with pcDNA3.1-*RP5-998N21.4* and then sonicated using a Qsonica instrument (USA). A total of ten biotinylated tilling probes were employed to capture lncRNA-*RP5-998N21.4* by hybridization with the sonicated chromatin in two separate reactions: one for odd-number probes (#1, 3, 5, 7, and 9) and another for even-numbered probes (#2, 4, 6, 8, and 10) synthesized by RiboBio technologies (RiboBio, China). The chromatin complexes associated with lncRNA-*RP5-998N21.4* were pulled down using streptavidin-conjugated magnetic beads and used for RNA and bound DNA isolation. The levels of lncRNA-*RP5-998N21.4* obtained from isolated RNA were quantified by qRT-PCR. The levels of bound *IFIT2* and *IFIT3* DNA from isolated DNAs were quantified by qPCR using primers in the predicted *IFIT2* and *IFIT3*-binding regions.

### RIP assay

RIP experiments were performed in cell extracts isolated from HEK 293 T cells transfected with pcDNA3.1-*RP5-998N21.4*. Nuclear extracts were immunoprecipitated with 5 µg of an anti-RBM14 antibody (Abcam, England) or isotype-matched control IgG overnight. RNA-protein-antibody complexes were captured using Protein A/G Dynabeads (Merck). RNA was eluted in accordance with the manufacturer’s instructions. cDNA was synthesized from eluted RNA using a HiScript 1st Strand cDNA Synthesis Kit (Vazyme, China) and analyzed by qPCR.

### Reporting summary

Further information on research design is available in the Nature Research Reporting Summary linked to this article.

## Supplementary information

Supplementary Information

Supplementary Data 1

Supplementary Data 2

Supplementary Data 3

Supplementary Data 4

Supplementary Data 5

Supplementary Data 6

Reporting Summary

## Data Availability

All data generated in the study are included in the article or uploaded as supplementary materials. RNA-Sequencing data of *RP5-998N21.4* overexpressed SK-N-SH cells have been deposited to the NCBI GEO site with the GEO number GSE186598. The source data of Fig. 3b are provided in Supplementary Data 5 and 6. Additional information is also available upon reasonable request to the corresponding authors.

## Code availability

Code used for data processing and analysis is available on request from the corresponding author.

## References

[1] Millan MJ (2016). Altering the course of schizophrenia: progress and perspectives. Nat. Rev. Drug Discov..

[2] Van Os,J, Kenis G, Rutten BP (2010). The environment and schizophrenia. Nature.

[3] Feigenson KA, Kusnecov AW, Silverstein SM (2014). Inflammation and the two-hit hypothesis of schizophrenia. Neurosci. Biobehav. Rev..

[4] Heyn H (2014). A symbiotic liaison between the genetic and epigenetic code. Front. Genet..

[5] Chew CL, Conos SA, Unal B, Tergaonkar V (2018). Noncoding RNAs: master regulators of inflammatory signaling. Trends Mol. Med..

[6] Lin, H. et al. The long noncoding RNA Lnczc3h7a promotes a TRIM25-mediated RIG-I antiviral innate immune response. *Nat. Immunol.* (2019). 10.1038/s41590-019-0379-0 31036902

[7] Mathy NW, Chen XM (2017). Long non-coding RNAs (lncRNAs) and their transcriptional control of inflammatory responses. J. Biol. Chem..

[8] Zhao, L. et al. A long non-coding RNA IVRPIE promotes host antiviral immune responses through regulating interferon β1 and ISG expression. *Front. Microbiol.* **11**, 2382 (2020). 10.3389/fmicb.2020.00260 PMC7044153 32153544

[9] Imaizumi T (2016). Interferon-stimulated gene (ISG) 60, as well as ISG56 and ISG54, positively regulates TLR3/IFN-beta/STAT1 axis in U373MG human astrocytoma cells. Neurosci. Res..

[10] Ni, C. et al. LncRNA-AC006129.1 reactivates a SOCS3-mediated anti-inflammatory response through DNA methylation-mediated CIC downregulation in schizophrenia. *Mol Psychiatry*10.1038/s41380-020-0662-3 (2020). 10.1038/s41380-020-0662-3 32015466

[11] Fromer M (2016). Gene expression elucidates functional impact of polygenic risk for schizophrenia. Nat. Neurosci..

[12] Gandal, M. J. et al. Transcriptome-wide isoform-level dysregulation in ASD, schizophrenia, and bipolar disorder. *Science* **362**, 1265 (2018). 10.1126/science.aat8127 PMC6443102 30545856

[13] Tian T (2018). The long noncoding RNA landscape in amygdala tissues from schizophrenia patients. EBioMedicine.

[14] Consortium GT (2017). Genetic effects on gene expression across human tissues. Nature.

[15] Fang S (2018). NONCODEV5: a comprehensive annotation database for long non-coding RNAs. Nucleic Acids Res..

[16] Mas-Ponte D (2017). LncATLAS database for subcellular localization of long noncoding RNAs. RNA.

[17] Zhou X (2013). Interferon induced IFIT family genes in host antiviral defense. Int. J. Biol. Sci..

[18] Xu F (2019). NF-kappaB-dependent IFIT3 induction by HBx promotes hepatitis B virus replication. Front. Microbiol..

[19] Duan J (2018). Transcriptomic signatures of schizophrenia revealed by dopamine perturbation in an ex vivo model. Transl. Psychiatry.

[20] Junker H (2007). Proteomic identification of an upregulated isoform of annexin A3 in the rat brain following reversible cerebral ischemia. Glia.

[21] Chong KWY (2010). Annexin A3 is associated with cell death in lactacystin-mediated neuronal injury. Neurosci. Lett..

[22] Wu X-M (2018). MicroRNA-339-3p alleviates inflammation and edema and suppresses pulmonary microvascular endothelial cell apoptosis in mice with severe acute pancreatitis-associated acute lung injury by regulating Anxa3 via the Akt/mTOR signaling pathway. J. Cell. Biochem..

[23] min X-l (2020). miR-18b attenuates cerebral ischemia/reperfusion injury through regulation of ANXA3 and PI3K/Akt signaling pathway. Brain Res. Bull..

[24] Yang C (2018). LncADeep: an ab initio lncRNA identification and functional annotation tool based on deep learning. Bioinformatics.

[25] Simon NE, Yuan M, Kai M (2017). RNA-binding protein RBM14 regulates dissociation and association of non-homologous end joining proteins. Cell Cycle.

[26] Muller N (2018). Inflammation in schizophrenia: pathogenetic aspects and therapeutic considerations. Schizophr. Bull..

[27] Sanders AR (2017). Transcriptome sequencing study implicates immune-related genes differentially expressed in schizophrenia: new data and a meta-analysis. Transl. Psychiatry.

[28] Jang Y (2020). Intrinsically disordered protein RBM14 plays a role in generation of RNA:DNA hybrids at double-strand break sites. Proc. Natl Acad. Sci. USA.

[29] Beyleveld, G. et al. Nucleolar relocalization of RBM14 by influenza A virus NS1 protein. *mSphere* **3**, e00549-18 (2018). 10.1128/mSphereDirect.00549-18 PMC6236804 30429226

[30] Ashley, C. L., Abendroth, A., McSharry, B. P. & Slobedman, B. Interferon-independent upregulation of interferon-stimulated genes during human cytomegalovirus infection is dependent on IRF3 expression. *Viruses* **11**, 246 (2019). 10.3390/v11030246 PMC6466086 30871003

[31] Richard MD, Brahm NC (2012). Schizophrenia and the immune system: pathophysiology, prevention, and treatment. Am. J. Health Syst. Pharm..

[32] Drexhage RC (2011). An activated set point of T-cell and monocyte inflammatory networks in recent-onset schizophrenia patients involves both pro- and anti-inflammatory forces. Int J. Neuropsychopharmacol..

[33] Jones KA, Thomsen C (2013). The role of the innate immune system in psychiatric disorders. Mol. Cell Neurosci..

[34] Kirkpatrick B, Miller BJ (2013). Inflammation and schizophrenia. Schizophr. Bull..

[35] Liu Y (2018). Non-coding RNA dysregulation in the amygdala region of schizophrenia patients contributes to the pathogenesis of the disease. Transl. Psychiatry.

[36] Langfelder P, Horvath S (2008). WGCNA: an R package for weighted correlation network analysis. BMC Bioinforma..

[37] Wang J, Duncan D, Shi Z, Zhang B (2013). WEB-based GEne SeT AnaLysis Toolkit (WebGestalt): update 2013. Nucleic Acids Res..

[38] Tiscornia G, Singer O, Verma IM (2006). Production and purification of lentiviral vectors. Nat. Protoc..

