# Supplementary material for: LncRNA RP5-998N21.4 promotes immune defense through upregulation of IFIT2 and IFIT3 in schizophrenia
Source: Schizophrenia (Heidelb). 2022 Mar 1;8(1):11. doi: 10.1038/s41537-021-00195-8 (PMC8888552; doi:10.1038/s41537-021-00195-8)
Supplement: Supplementary file 1 — Supplementary Information [file 41537_2021_195_MOESM1_ESM.pdf]

SUPPLEMENTARY FIGURES

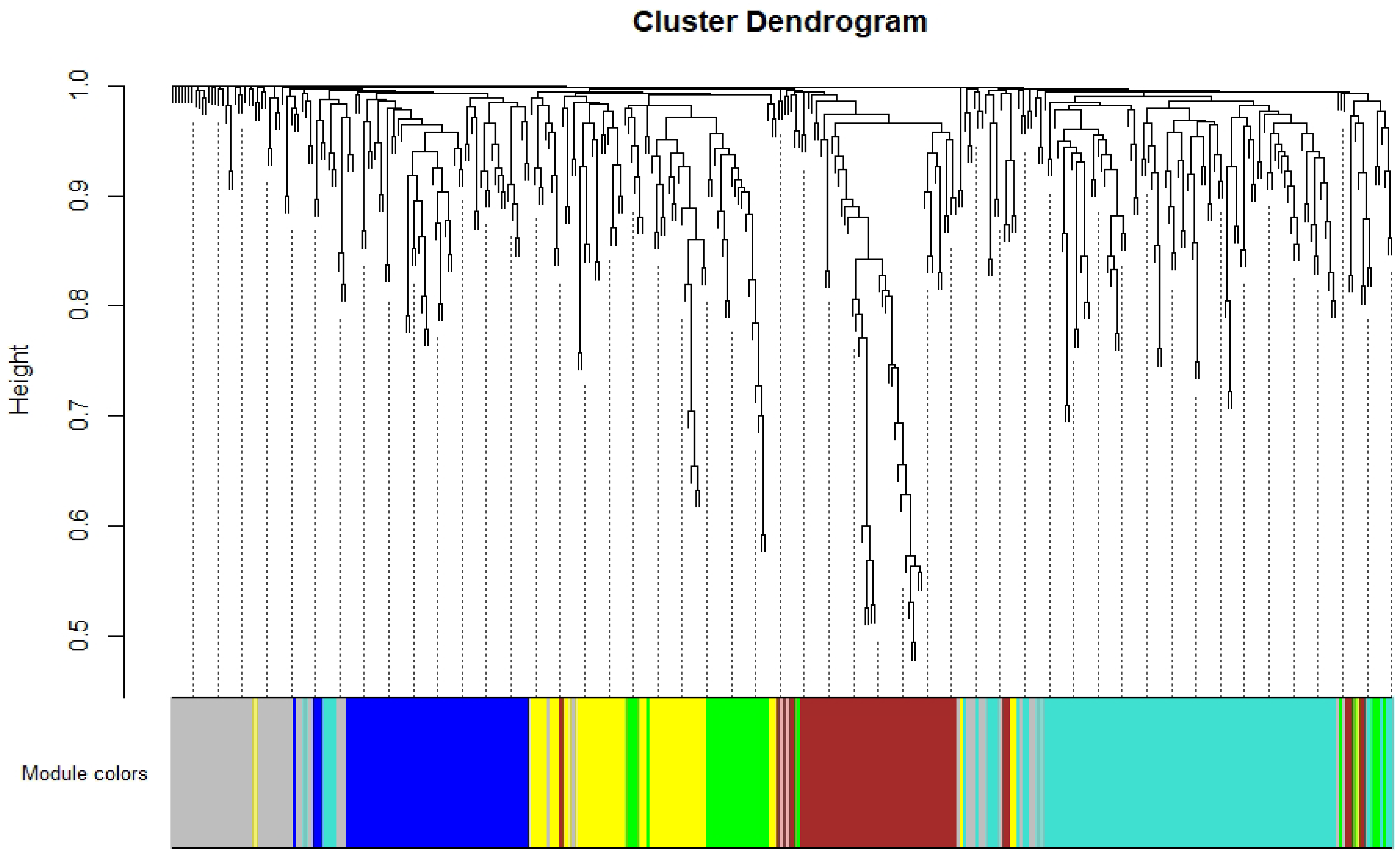

Supplementary Figure 1 Hierarchical cluster of module's eigengenes co-expression network (top) and the topological overlap matrix (TOM) plot (bottom) for lncRNA-*RP5-998N21.4* and its correlated mRNAs. The rows and columns represent the same set of genes sorted by the hierarchical clustering tree of TOM with modules represented by colored labels.

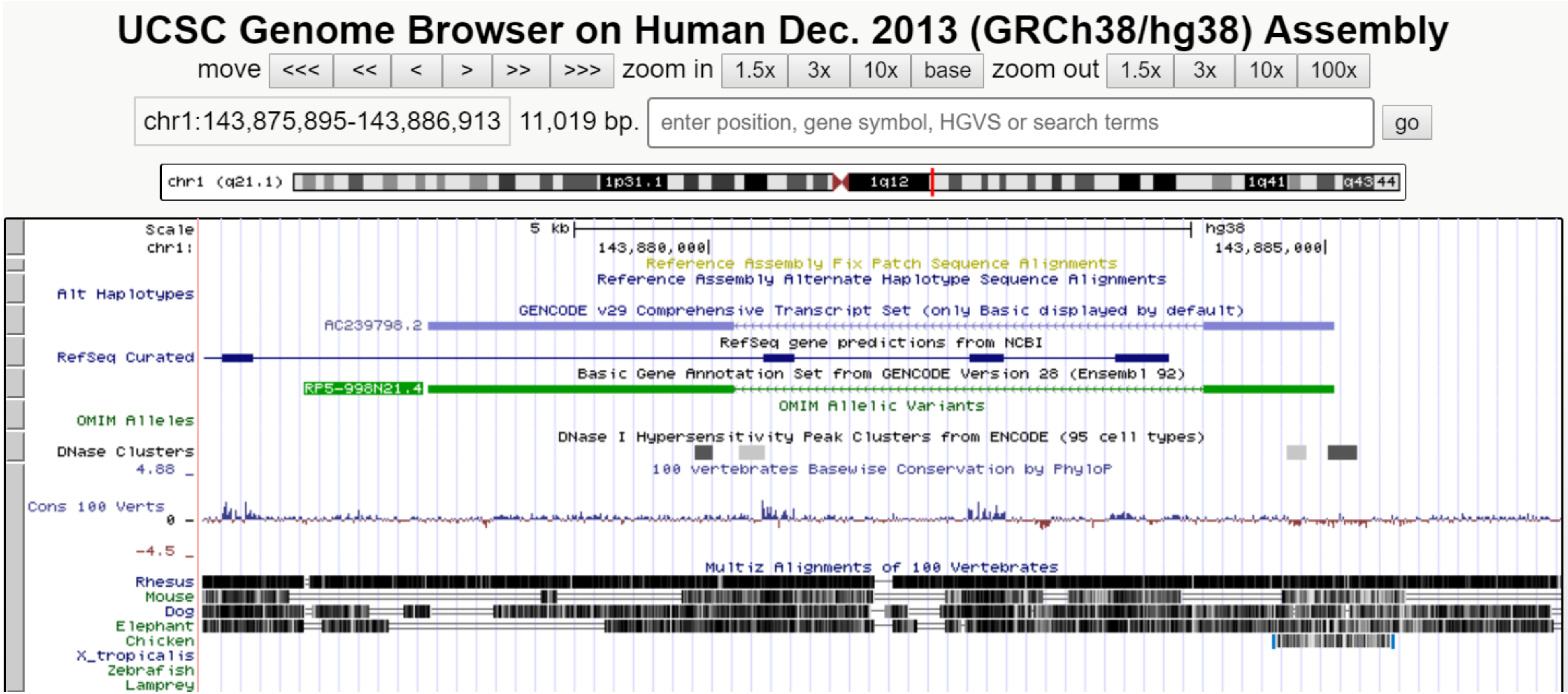

Supplementary Figure 2 Annotations of lncRNA-*RP5-998N21.4* shown in the UCSC database.

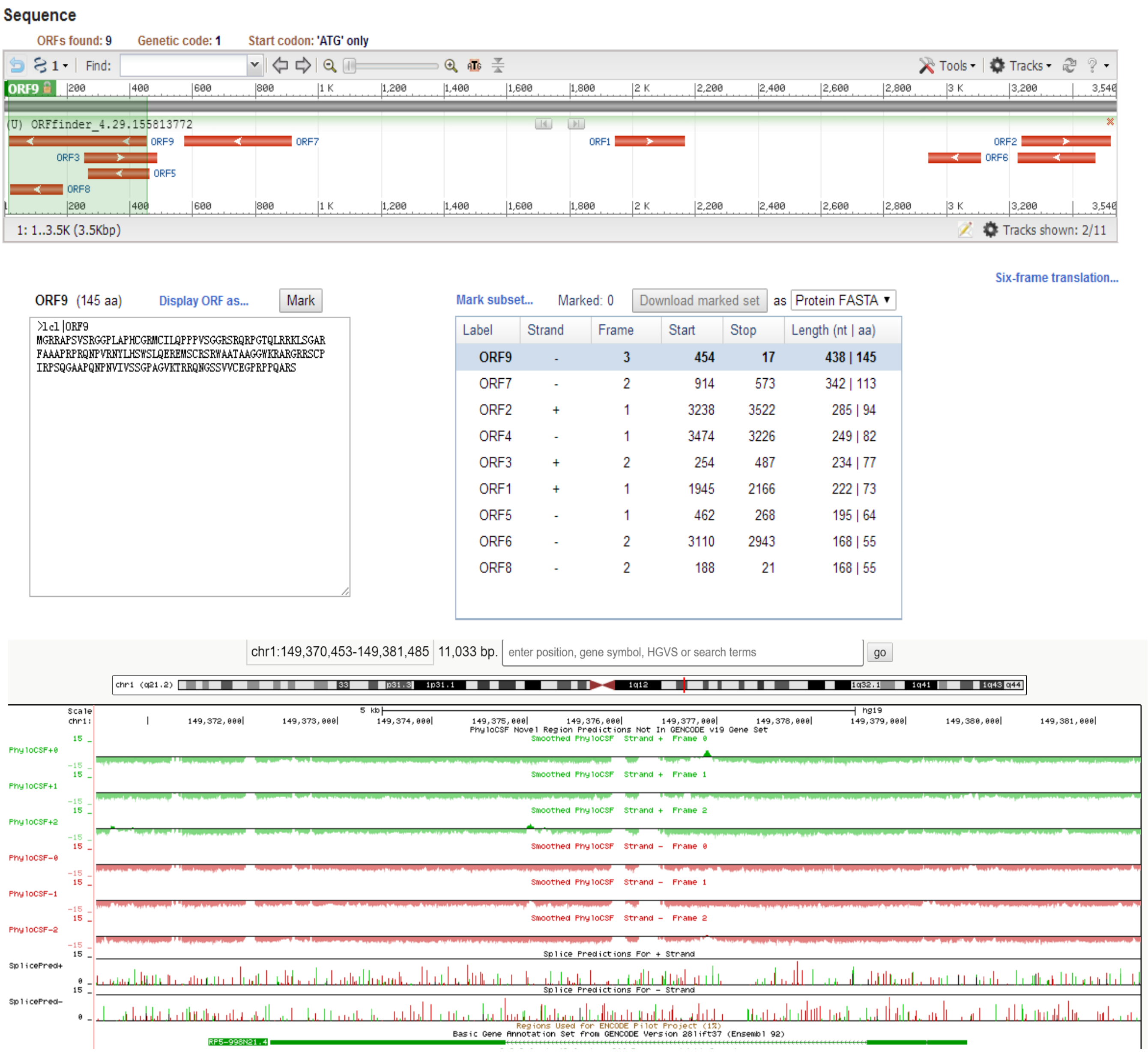

Label	Strand	Frame	Start	Stop	Length (nt | aa)
ORF9	-	3	454	17	438 | 145
ORF7	-	2	914	573	342 | 113
ORF2	+	1	3238	3522	285 | 94
ORF4	-	1	3474	3226	249 | 82
ORF3	+	2	254	487	234 | 77
ORF1	+	1	1945	2166	222 | 73
ORF5	-	1	462	268	195 | 64
ORF6	-	2	3110	2943	168 | 55
ORF8	-	2	188	21	168 | 55

Supplementary Figure 3 *In silico* results showing the protein-coding ability of *AC006129.1* and *RP5-998N21.4* obtained using the ORFfinder and Phylogenetic Codon Substitution Frequency tools.

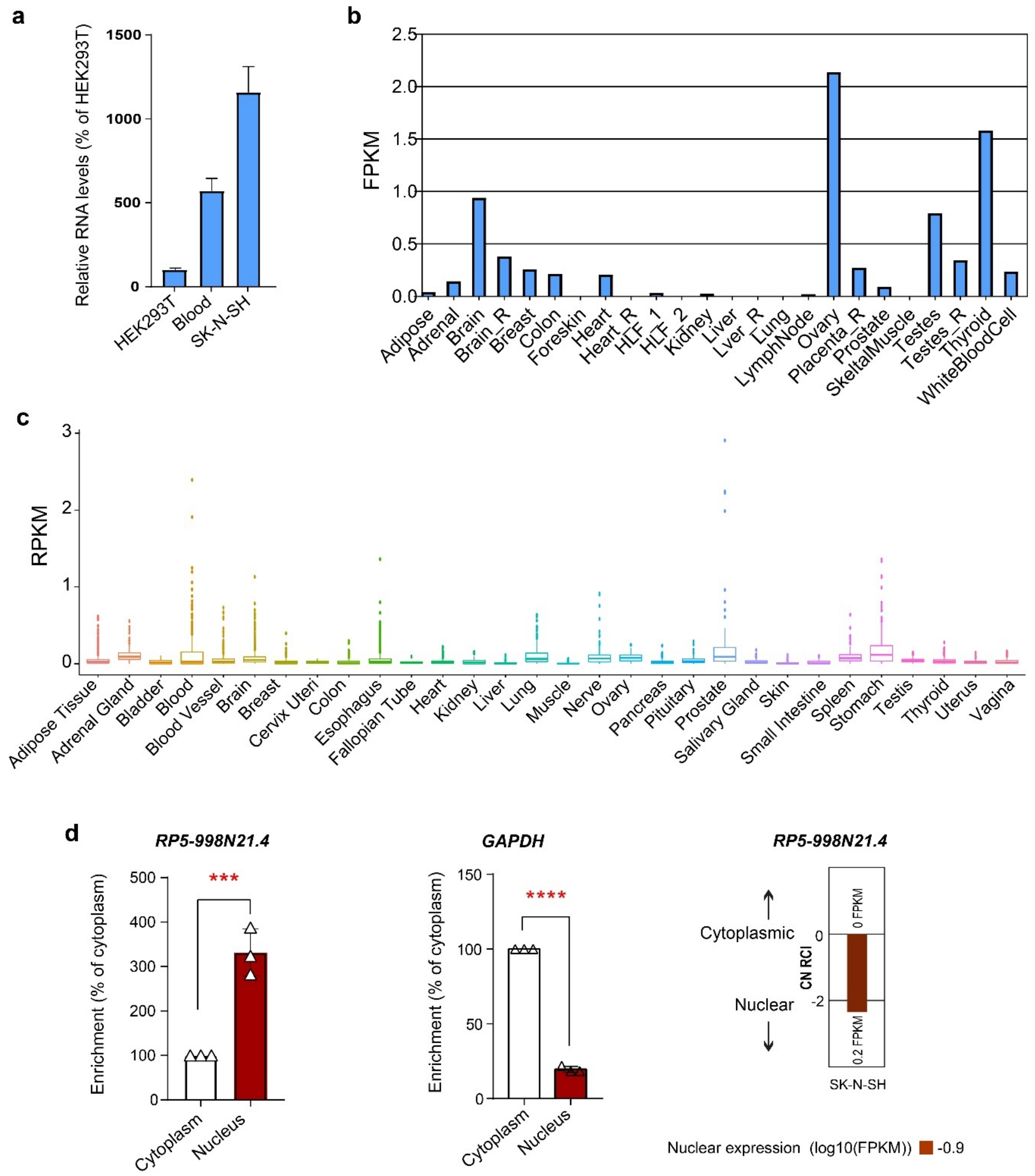

Supplementary Figure 4 Subcellular localization analysis of *RP5-998N24.1* (**a**) qRT-PCR analysis of *RP5-998N24.1* level in HEK293T cells, human white blood cells, and SK-N-SH cells. (**b-c**) Tissue expression patterns of *RP5-998N24.1* from the Noncode (**http://www.noncode.org/keyword.php**, **b**) or GTEx (**https://www.gtexportal.org/home/index.html**, **c**) data sets. (**d**) The subcellular localization of *RP5-998N24.1* was extraordinarily nuclear rather than cytoplasmic, as determined by qPCR analysis of HEK293T cells (left) and RNA-seq data from SK-N-SH from the lncATLAS dataset (right; **http://lncatlas.crg.eu**). The data from each point are shown as the mean ± s.d. values.

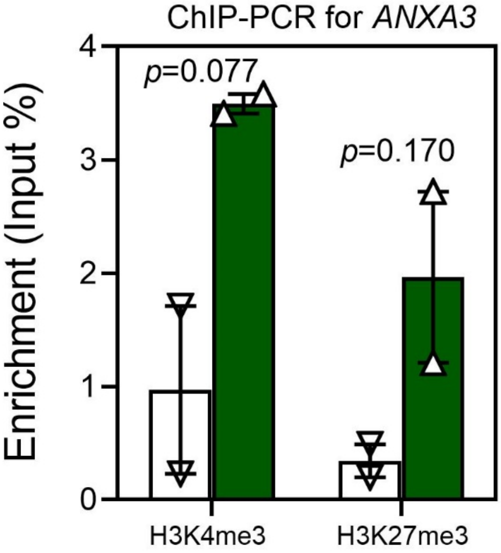

Supplementary Figure 5 ChIP-qPCR analysis of H3K4me3 and H3K27me3 enrichments at the promoter regions of *ANXA3* in HEK293T cells with or without the *RP5-998N21.4*$_{OE}$. The data from each point are shown as the mean ± s.d. values.

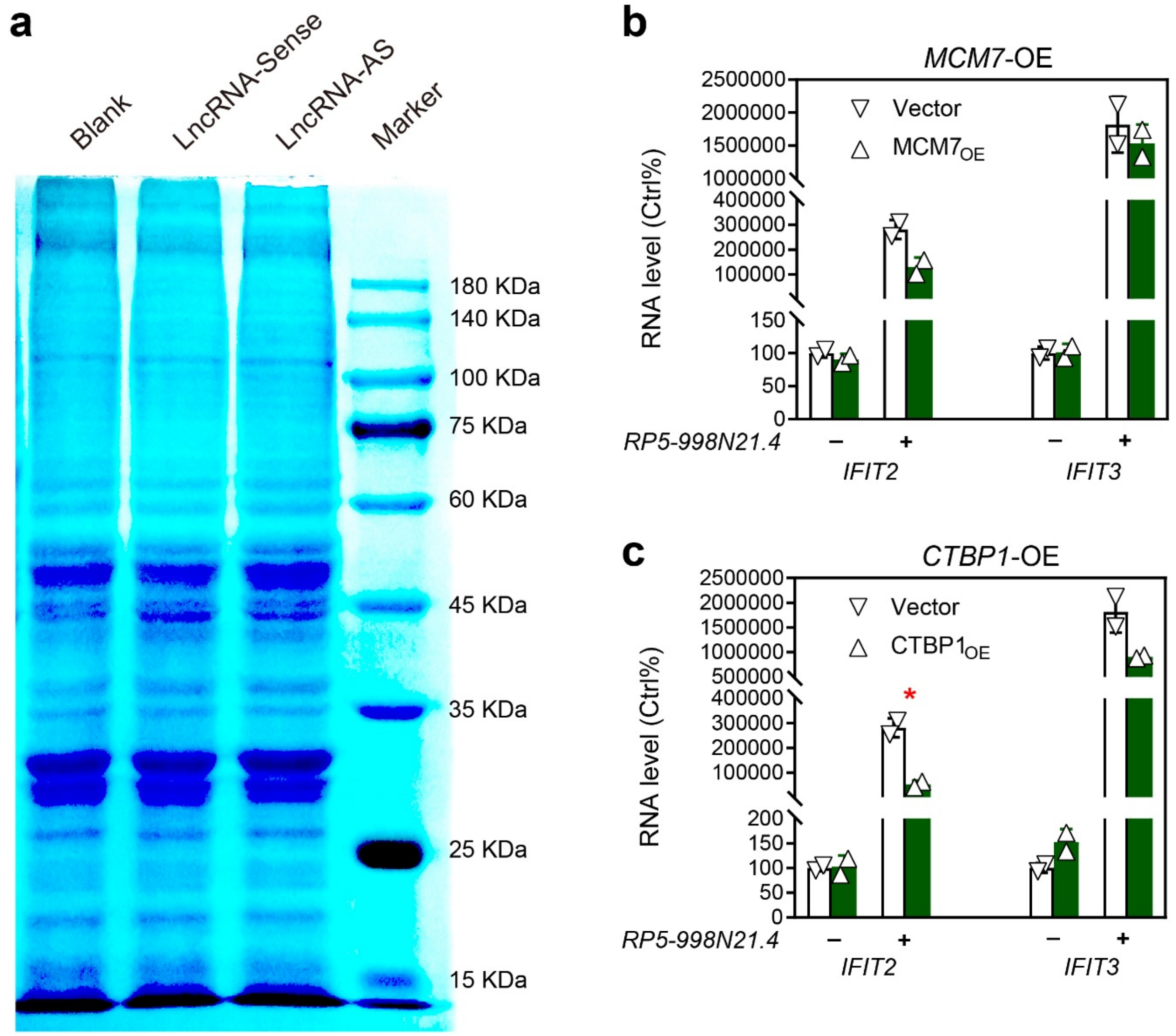

Supplementary Figure 6 Coomassie Brilliant Blue staining of the proteins pulled down by *PR5-998N21.4* from HEK293T cell lysates (**a**) and effects of the *RP5-998N21.4*$_{OE}$ on the mRNA levels of *IFIT2* and *IFIT3* in HEK293T cells with or without *MCM7*$_{\mathrm{OE}}$ (**b**) or *CTBP1*$_{OE}$ (**c**). The data from each point are shown as the mean ± s.d. values.

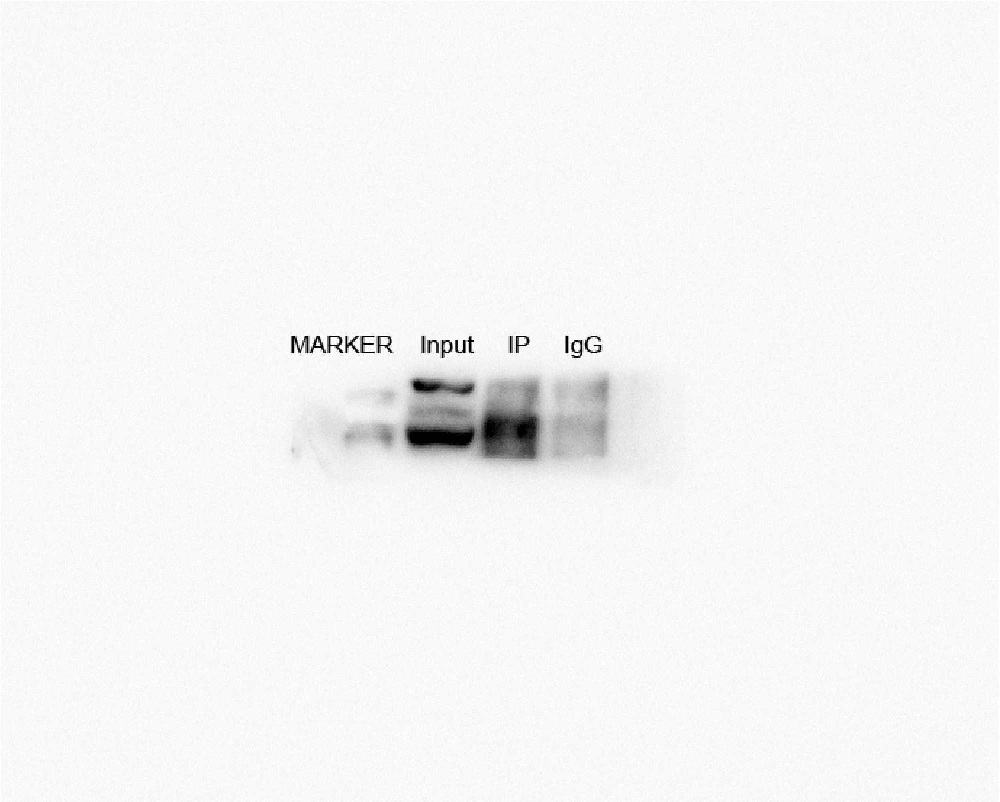

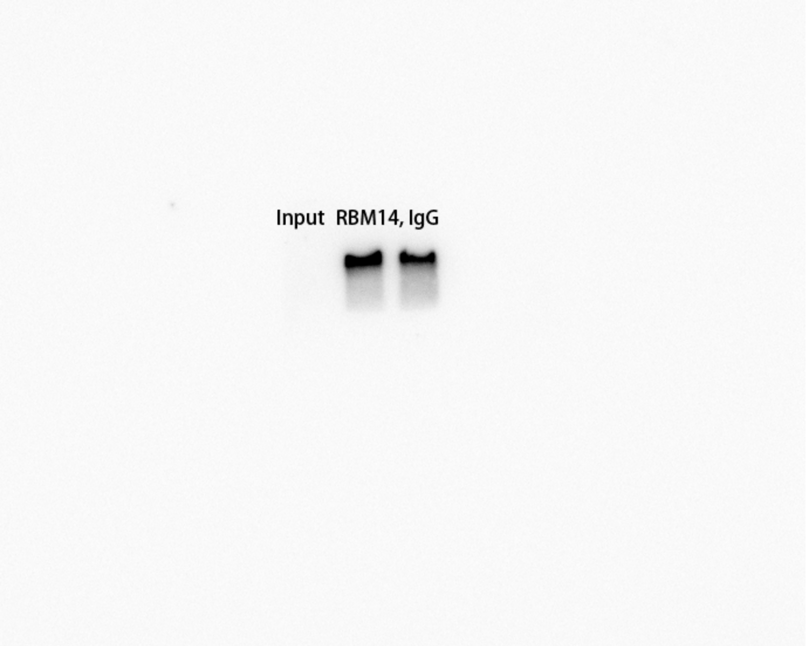

Supplementary Figure 7 The un-cropped images for immunoprecipitation (IP) blots of RNA with the anti-RMB14 antibody (**upper,** marker for 75 kDa and 60 kDa) and immunoglobin G (IgG) served as the negative immunoprecipitation control (**lower**) in HEK293T cells.

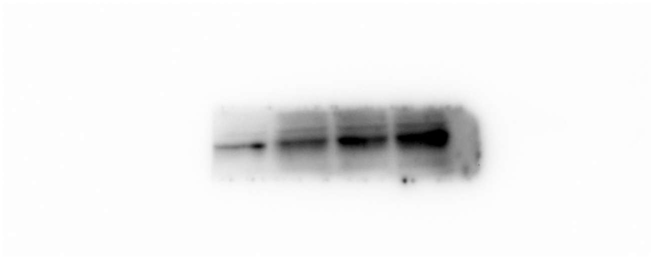

IFIT2

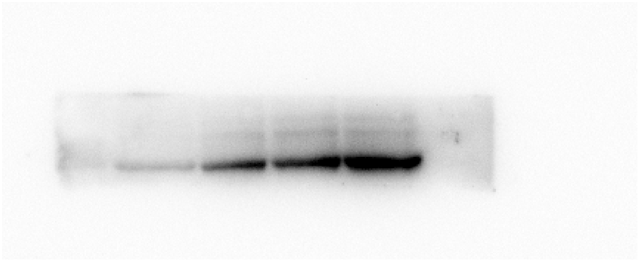

IFIT3

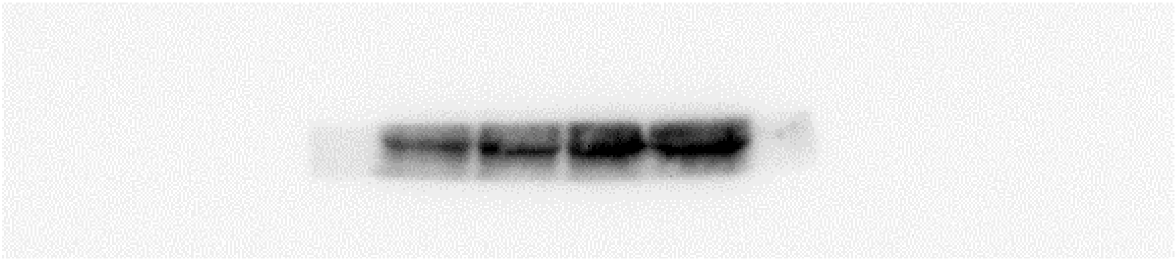

p-STAT1

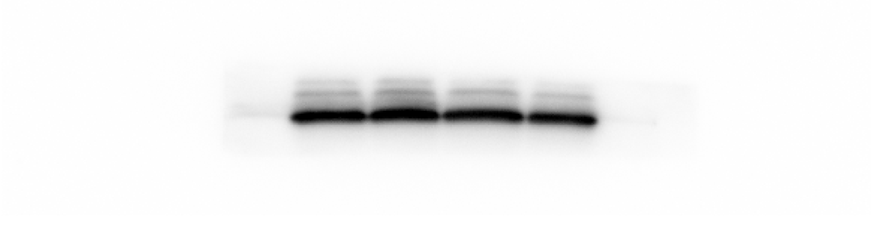

GAPDH

Supplementary Figure 8 The un-cropped images of immunoblot shown in Figure 4a

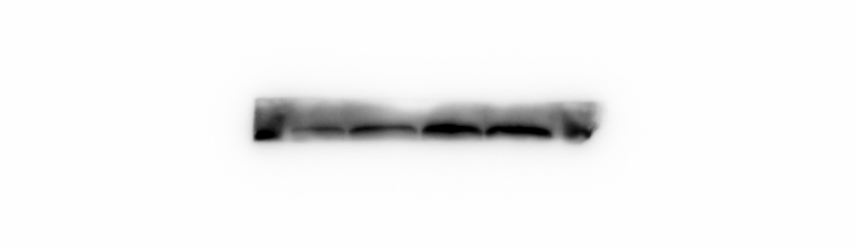

IFIT2

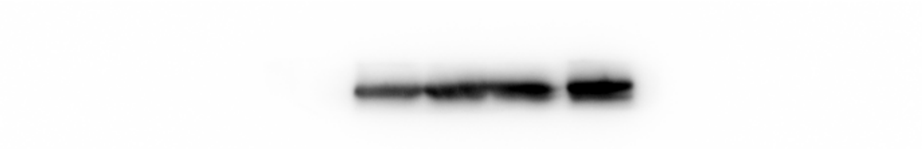

IFIT3

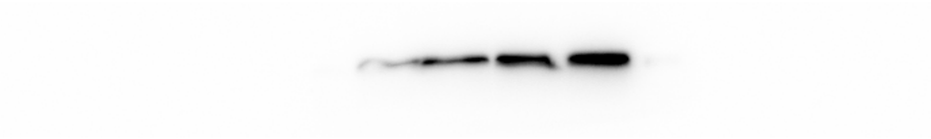

p-STAT1

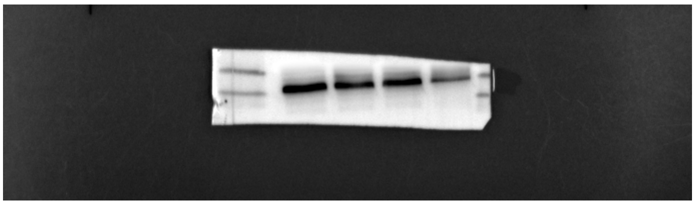

STAT1

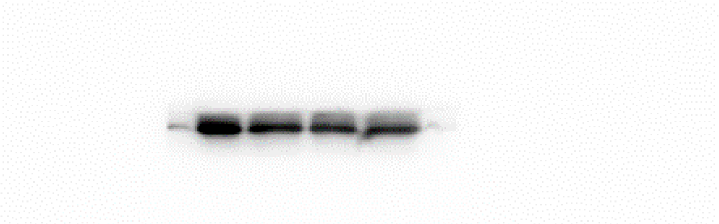

GAPDH

Supplementary Figure 9 The un-cropped images of immunoblot shown in Figure 4b

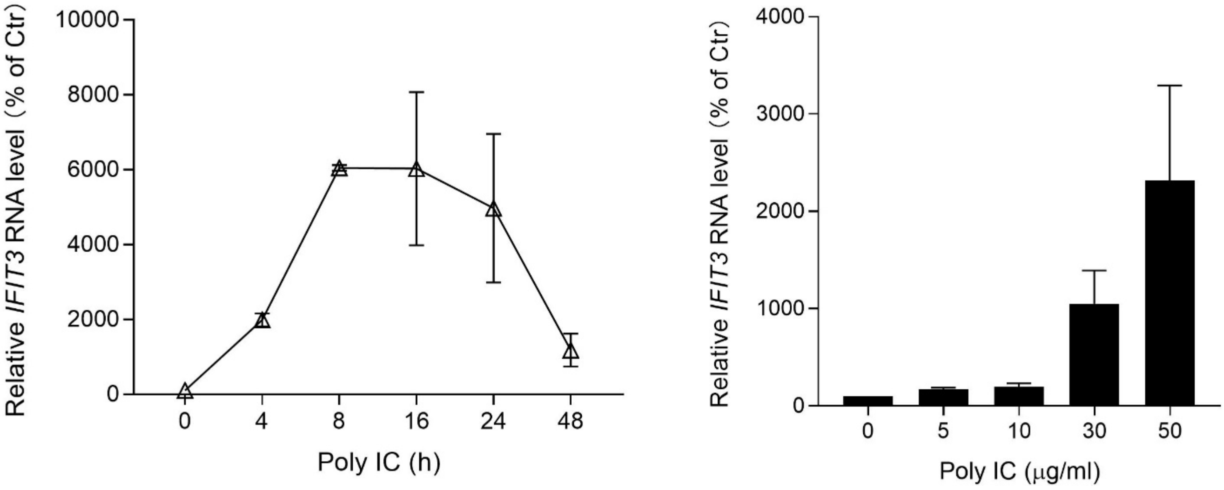

Supplementary Figure 10 Time-dependent (left) and dose-dependent (right) expression patterns of *IFIT2* RNA level induced by poly IC in U-251 cells. **(Left)** U-251 cells were cultured and treated with 50 μg/ml of poly IC for up to 48 h. **(Right)** The cells were treated with various concentrations of poly IC for 4 h. The data from each point are shown as the mean ± s.d. values.

SUPPLEMENTARY TABLES

Supplementary Table 1 Information of lncRNA *RP5-998N21.4-correlated 367 mRNAs*

**ID**	**SYMBOL**	**GENE_POSITION.X**
ENSG00000004777	*ARHGAP33*	CHR19:36265433-36279724
ENSG00000127124	*HIVEP3*	CHR1:41972035-42501596
ENSG00000176533	*GNG7*	CHR19:2511216-2702707
ENSG00000167483	*FAM129C*	CHR19:17634109-17664648
ENSG00000079313	*REXO1*	CHR19:1815247-1848452
ENSG00000251606	*CTD-2215E18.1*	CHR5:96424778-96772921
ENSG00000205784	*ARRDC5*	CHR19:4890448-4902879
ENSG00000008710	*PKD1*	CHR16:2089815-2185899
ENSG00000181036	*FCRL6*	CHR1:159770300-159786041
ENSG00000159674	*SPON2*	CHR4:1160719-1202750
ENSG00000012124	*CD22*	CHR19:35810163-35838258
ENSG00000107902	*LHPP*	CHR10:126150402-126480439
ENSG00000090924	*PLEKHG2*	CHR19:39903224-39919055
ENSG00000128394	*APOBEC3F*	CHR22:39436608-39483748
ENSG00000163534	*FCRL1*	CHR1:157764192-157789895
ENSG00000069702	*TGFBR3*	CHR1:92145901-92371892
ENSG00000087086	*FTL*	CHR19:49468557-49470135
ENSG00000119922	*IFIT2*	CHR10:90973325-91174314

ENSG00000164104	*HMGB2*	CHR4:174252845-174256276
ENSG00000188987	*HIST1H4D*	CHR6:26188937-26189304
ENSG00000103569	*AQP9*	CHR15:58245621-58861151
ENSG00000135940	*COX5B*	CHR2:98262502-98264846
ENSG00000108244	*KRT23*	CHR17:39077681-39132178
ENSG00000180596	*HIST1H2BC*	CHR6:26115100-26124154
ENSG00000163421	*PROK2*	CHR3:71820806-71834357
ENSG00000145491	*ROPN1L*	CHR5:10441401-10472141
ENSG00000157551	*KCNJ15*	CHR21:39493544-39673748
ENSG00000176788	*BASP1*	CHR5:17217668-17276943
ENSG00000169385	*RNASE2*	CHR14:21423610-21424595
ENSG00000137752	*CASP1*	CHR11:104896169-104972158
ENSG00000189068	*VSTM1*	CHR19:54544078-54567207
ENSG00000196954	*CASP4*	CHR11:104813592-104840163
ENSG00000102524	*TNFSF13B*	CHR13:108903587-108960832
ENSG00000124529	*HIST1H4B*	CHR6:26027123-26027480
ENSG00000197208	*SLC22A4*	CHR5:131520568-131731306
ENSG00000151726	*ACSL1*	CHR4:185676748-185747972
ENSG00000163235	*TGFA*	CHR2:70674411-70781325
ENSG00000073737	*DHRS9*	CHR2:169921298-169952677
ENSG00000173281	*PPP1R3B*	CHR8:8993764-9009084

ENSG00000121858	*TNFSF10*	CHR3:172223297-172241297
ENSG00000171236	*LRG1*	CHR19:4522542-4540486
ENSG00000115271	*GCA*	CHR2:163175349-163695240
ENSG00000170837	*GPR27*	CHR3:71728439-71805647
ENSG00000183621	*ZNF438*	CHR10:31133562-31320866
ENSG00000119917	*IFIT3*	CHR10:90973325-91174314
ENSG00000138678	*AGPAT9*	CHR4:84457066-84527028
ENSG00000162747	*FCGR3B*	CHR1:161511548-161648444
ENSG00000204397	*CARD16*	CHR11:104896169-104972158
ENSG00000113368	*LMNB1*	CHR5:126112314-126172712
ENSG00000256812	*CAPNS2*	CHR16:55542909-55620582
ENSG00000197238	*HIST1H4J*	CHR6:27791883-27792257
ENSG00000136810	*TXN*	CHR9:113006090-113018920
ENSG00000198558	*HIST1H4L*	CHR6:27840925-27841289
ENSG00000136514	*RTP4*	CHR3:187086119-187089864
ENSG00000112303	*VNN2*	CHR6:133065008-133084598
ENSG00000196890	*HIST3H2BB*	CHR1:228645807-228646259
ENSG00000182117	*NOP10*	CHR15:34633916-34635378
ENSG00000143546	*S100A8*	CHR1:153362507-153363664
ENSG00000120217	*CD274*	CHR9:5450502-5470566
ENSG00000188313	*PLSCR1*	CHR3:146232966-146262651

ENSG00000090382	*LYZ*	CHR12:69742120-69748014
ENSG00000163568	*AIM2*	CHR1:159032273-159116886
ENSG00000139832	*RAB20*	CHR13:111175416-111214080
ENSG00000123610	*TNFAIP6*	CHR2:152214105-152236560
ENSG00000148926	*ADM*	CHR11:10326226-10328944
ENSG00000198019	*FCGR1B*	CHR1:120906027-120935937
ENSG00000257017	*HP*	CHR16:72078187-72210777
ENSG00000184557	*SOCS3*	CHR17:76352863-76356158
ENSG00000148346	*LCN2*	CHR9:130911349-130915734
ENSG00000163220	*S100A9*	CHR1:153330329-153333503
ENSG00000138772	*ANXA3*	CHR4:79472672-79531597
ENSG00000150337	*FCGR1A*	CHR1:149754226-149783928
ENSG00000121552	*CSTA*	CHR3:122044090-122060819
ENSG00000149516	*MS4A3*	CHR11:59824059-59838601
ENSG00000179869	*ABCA13*	CHR7:48211054-48687092
ENSG00000163221	*S100A12*	CHR1:153346183-153348125
ENSG00000152766	*ANKRD22*	CHR10:90581888-90611575
ENSG00000096006	*CRISP3*	CHR6:49695096-49712150
ENSG00000134827	*TCN1*	CHR11:59620272-59634048
ENSG00000164047	*CAMP*	CHR3:48264836-48266981
ENSG00000204936	*CD177*	CHR19:43857824-43867480

ENSG00000009694	*TENM1*	CHRX:123093082-124097666
ENSG00000178209	*PLEC*	CHR8:144989320-145050902
ENSG00000185989	*RASA3*	CHR13:114747193-114898086
ENSG00000178921	*PFAS*	CHR17:8130190-8173809
ENSG00000100027	*YPEL1*	CHR22:22006558-22090123
ENSG00000204852	*TCTN1*	CHR12:111051831-111142755
ENSG00000108262	*GIT1*	CHR17:27887564-28513493
ENSG00000014138	*POLA2*	CHR11:65029232-65073060
ENSG00000100083	*GGA1*	CHR22:38004480-38029571
ENSG00000255221	*CARD17*	CHR11:104896169-104972158
ENSG00000172197	*MBOAT1*	CHR6:20100934-20294706
ENSG00000241553	*ARPC4*	CHR3:9791627-9896822
ENSG00000017621	*MAGIX*	CHRX:49019060-49024822
ENSG00000143753	*DEGS1*	CHR1:224363457-224381143
ENSG00000132581	*SDF2*	CHR17:26975373-27029697
ENSG00000128335	*APOL2*	CHR22:36622255-36636000
ENSG00000182831	*C16ORF72*	CHR16:9185504-9215497
ENSG00000196743	*GM2A*	CHR5:150560612-150650001
ENSG00000144118	*RALB*	CHR2:120997639-121052289
ENSG00000146425	*DYNLT1*	CHR6:159057505-159065771
ENSG00000157800	*SLC37A3*	CHR7:139993492-140126050

ENSG00000168288	*MMADHC*	CHR2:150426147-150715705
ENSG00000132963	*POMP*	CHR13:29233240-29253062
ENSG00000097033	*SH3GLB1*	CHR1:87170258-87213867
ENSG00000123609	*NMI*	CHR2:152126978-152146571
ENSG00000177674	*AGTRAP*	CHR1:11796140-11814859
ENSG00000058091	*CDK14*	CHR7:89964536-90839905
ENSG00000135828	*RNASEL*	CHR1:182542768-182558391
ENSG00000166946	*CCNDBP1*	CHR15:43398422-43513481
ENSG00000136689	*IL1RN*	CHR2:113864790-113891593
ENSG00000131100	*ATP6V1E1*	CHR22:18074901-18111584
ENSG00000125538	*IL1B*	CHR2:113587327-113594480
ENSG00000111144	*LTA4H*	CHR12:96390298-96437298
ENSG00000159479	*MED8*	CHR1:43849587-43855479
ENSG00000118564	*FBXL5*	CHR4:15606161-15739936
ENSG00000138496	*PARP9*	CHR3:122246770-122294050
ENSG00000129355	*CDKN2D*	CHR19:10677137-10679735
ENSG00000196226	*HIST1H2BB*	CHR6:26043454-26043885
ENSG00000253276	*CCDC71L*	CHR7:106012696-106410653
ENSG00000158869	*FCER1G*	CHR1:161185023-161193421
ENSG00000129204	*USP6*	CHR17:5008835-5078329
ENSG00000163823	*CCR1*	CHR3:46205095-46308197

ENSG00000198734	*F5*	CHR1:169483403-169555826
ENSG00000167207	*NOD2*	CHR16:50727513-50766988
ENSG00000143226	*FCGR2A*	CHR1:161475219-161493803
ENSG00000109270	*LAMTOR3*	CHR4:100799492-100815647
ENSG00000058799	*YIPF1*	CHR1:54317391-54356407
ENSG00000145365	*TIFA*	CHR4:113196782-113363776
ENSG00000164032	*H2AFZ*	CHR4:100869242-101136662
ENSG00000101425	*BPI*	CHR20:36838889-36965907
ENSG00000180953	*ST20*	CHR15:80125926-80217194
ENSG00000025039	*RRAGD*	CHR6:90074354-90121989
ENSG00000217555	*CKLF*	CHR16:66586465-66613040
ENSG00000112062	*MAPK14*	CHR6:35995487-36079013
ENSG00000120656	*TAF12*	CHR1:28915834-28969597
ENSG00000162645	*GBP2*	CHR1:89571814-89641723
ENSG00000175471	*MCTP1*	CHR5:93488670-94620279
ENSG00000203747	*FCGR3A*	CHR1:161511548-161648444
ENSG00000072952	*MRVI1*	CHR11:10562818-10715535
ENSG00000155926	*SLA*	CHR8:133879202-134147147
ENSG00000132825	*PPP1R3D*	CHR20:58508818-58523735
ENSG00000127954	*STEAP4*	CHR7:87834432-87936206
ENSG00000111052	*LIN7A*	CHR12:81186298-81650533

ENSG00000126945	*HNRNPH2*	CHRX:100645811-100669121
ENSG00000099860	*GADD45B*	CHR19:2476119-2478257
ENSG00000100504	*PYGL*	CHR14:51314839-51411454
ENSG00000187554	*TLR5*	CHR1:223282747-223316624
ENSG00000229644	*NAMPTL*	CHR10:36810648-36813162
ENSG00000165181	*C9ORF84*	CHR9:114448452-114557288
ENSG00000163754	*GYG1*	CHR3:148709127-148745419
ENSG00000140859	*KIFC3*	CHR16:57792128-57896957
ENSG00000270149	*F11R*	CHR1:160965000-161008784
ENSG00000198216	*CACNA1E*	CHR1:181382237-181777219
ENSG00000121931	*LRIF1*	CHR1:111486088-111506701
ENSG00000186827	*TNFRSF4*	CHR1:1146705-1149518
ENSG00000169612	*FAM103A1*	CHR15:83509837-83772195
ENSG00000106245	*BUD31*	CHR7:98923520-99063954
ENSG00000176531	*PHLDB3*	CHR19:43979105-44009087
ENSG00000092036	*HAUS4*	CHR14:23398817-23451851
ENSG00000111843	*TMEM14C*	CHR6:10723147-10731362
ENSG00000143933	*CALM2*	CHR2:47126413-47403740
ENSG00000174446	*SNAPC5*	CHR15:66679154-66790151
ENSG00000104884	*ERCC2*	CHR19:45836691-45874176
ENSG00000185909	*KLHDC8B*	CHR3:49209043-49213917

ENSG00000059145	*UNKL*	CHR16:1401923-1464752
ENSG00000121691	*CAT*	CHR11:34460471-34493609
ENSG00000204516	*MICB*	CHR6:31462657-31478901
ENSG00000136521	*NDUFB5*	CHR3:179322477-179345435
ENSG00000196968	*FUT11*	CHR10:75531934-75538821
ENSG00000106477	*CEP41*	CHR7:130036374-130082274
ENSG00000152213	*ARL11*	CHR13:50202434-50208008
ENSG00000156414	*TDRD9*	CHR14:104394798-104519004
ENSG00000174021	*GNG5*	CHR1:84964007-85040163
ENSG00000124107	*SLPI*	CHR20:43880879-43883205
ENSG00000118922	*KLF12*	CHR13:74260225-74708394
ENSG00000143543	*JTB*	CHR1:153931574-153950164
ENSG00000050130	*JKAMP*	CHR14:59895739-60043549
ENSG00000178860	*MSC*	CHR8:72740401-73030628
ENSG00000109046	*WSB1*	CHR17:25621101-25640657
ENSG00000070190	*DAPP1*	CHR4:100737989-100791311
ENSG00000198374	*HIST1H2AL*	CHR6:27833033-27833606
ENSG00000169714	*CNBP*	CHR3:128888326-128902765
ENSG00000137757	*CASP5*	CHR11:104864961-104893895
ENSG00000183019	*C19ORF59*	CHR19:7741513-7747744
ENSG00000124215	*CDH26*	CHR20:58533470-58609066

ENSG00000186522	*SEPTIN10*	CHR2:110300558-110371783
ENSG00000101558	*VAPA*	CHR18:9913998-9960018
ENSG00000153898	*MCOLN2*	CHR1:85391267-85462796
ENSG00000232119	*MCTS1*	CHRX:119727864-119754929
ENSG00000198648	*STK39*	CHR2:168810529-169104651
ENSG00000134152	*KATNBL1*	CHR15:34432874-34502297
ENSG00000125967	*NECAB3*	CHR20:32244892-32262269
ENSG00000258366	*RTEL1*	CHR20:62289162-62374858
ENSG00000101084	*C20ORF24*	CHR20:34894257-35274619
ENSG00000145817	*YIPF5*	CHR5:143537722-143550278
ENSG00000183696	*UPP1*	CHR7:48128224-48148330
ENSG00000177000	*MTHFR*	CHR1:11821843-11908402
ENSG00000118513	*MYB*	CHR6:135502452-135540311
ENSG00000175197	*DDIT3*	CHR12:57853917-57914300
ENSG00000183726	*TMEM50A*	CHR1:25568727-25756683
ENSG00000124469	*CEACAM8*	CHR19:42901279-43156507
ENSG00000118113	*MMP8*	CHR11:102582525-102597781
ENSG00000185504	*C17ORF70*	CHR17:79506910-79520987
ENSG00000183978	*COA3*	CHR17:40932695-40950722
ENSG00000197021	*CXORF40B*	CHRX:149097744-149392815
ENSG00000113811	*SELK*	CHR3:53918436-53926015

ENSG00000137414	*FAM8A1*	CHR6:17600585-17611950
ENSG00000112053	*SLC26A8*	CHR6:35911290-35992645
ENSG00000163840	*DTX3L*	CHR3:122246770-122294050
ENSG00000116793	*PHTF1*	CHR1:114239452-114302111
ENSG00000147604	*RPL7*	CHR8:74202505-74268696
ENSG00000111254	*AKAP3*	CHR12:4671369-4960277
ENSG00000133466	*C1QTNF6*	CHR22:37521877-37595425
ENSG00000122986	*HVCN1*	CHR12:111051831-111142755
ENSG00000101150	*TPD52L2*	CHR20:62496642-62522898
ENSG00000089289	*IGBP1*	CHRX:69353298-69386174
ENSG00000181038	*METTL23*	CHR17:74722911-74777531
ENSG00000099968	*BCL2L13*	CHR22:18111620-18213388
ENSG00000154518	*ATP5G3*	CHR2:176040985-176049335
ENSG00000102763	*VWA8*	CHR13:42140972-42535256
ENSG00000198805	*PNP*	CHR14:20937112-20945253
ENSG00000089123	*TASP1*	CHR20:13202417-13619587
ENSG00000164983	*TMEM65*	CHR8:125324230-125384933
ENSG00000123080	*CDKN2C*	CHR1:51426416-51440305
ENSG00000257743	*RP11-1220K2.2*	CHR7:141811548-141922124
ENSG00000100483	*METTL21D*	CHR14:50575349-50583318
ENSG00000172172	*MRPL13*	CHR8:121392999-121825513

ENSG00000169129	*AFAP1L2*	CHR10:116054582-116164515
ENSG00000163319	*MRPS18C*	CHR4:84377084-84444501
ENSG00000151470	*C4ORF33*	CHR4:129730778-130034487
ENSG00000198604	*BAZ1A*	CHR14:35221936-35345665
ENSG00000132664	*POLR3F*	CHR20:18364010-18465287
ENSG00000197846	*HIST1H2BF*	CHR6:26199747-26200942
ENSG00000120832	*MTERFD3*	CHR12:107349496-107380944
ENSG00000137133	*HINT2*	CHR9:35812956-35854844
ENSG00000213722	*DDAH2*	CHR6:31694814-31707540
ENSG00000012223	*LTF*	CHR3:46477135-46526724
ENSG00000143479	*DYRK3*	CHR1:206807993-206857764
ENSG00000124693	*HIST1H3B*	CHR6:26031816-26032288
ENSG00000115561	*CHMP3*	CHR2:86730553-87005164
ENSG00000127184	*COX7C*	CHR5:85913720-85916779
ENSG00000196235	*SUPT5H*	CHR19:39926795-39967310
ENSG00000155827	*RNF20*	CHR9:104296132-104325622
ENSG00000221937	*TAS2R40*	CHR7:142919129-142920162
ENSG00000155744	*FAM126B*	CHR2:201773695-201950473
ENSG00000164821	*DEFA4*	CHR8:6793343-6795860
ENSG00000133619	*KRBA1*	CHR7:149411871-149431664
ENSG00000165476	*REEP3*	CHR10:65281122-65384883

ENSG00000112343	*TRIM38*	CHR6:25963029-25985348
ENSG00000144566	*RAB5A*	CHR3:19988570-20053822
ENSG00000241343	*RPL36A*	CHRX:100645811-100669121
ENSG00000157734	*SNX22*	CHR15:64443913-64455404
ENSG00000226479	*TMEM185B*	CHR2:120978853-120980984
ENSG00000116205	*TCEANC2*	CHR1:54519259-54578192
ENSG00000069974	*RAB27A*	CHR15:55495163-55800432
ENSG00000138069	*RAB1A*	CHR2:65283499-65357240
ENSG00000116857	*TMEM9*	CHR1:201103899-201140702
ENSG00000184216	*IRAK1*	CHRX:153275950-153285440
ENSG00000114784	*EIF1B*	CHR3:40351174-40353915
ENSG00000169764	*UGP2*	CHR2:64068073-64118696
ENSG00000113916	*BCL6*	CHR3:187416046-187463515
ENSG00000196116	*TDRD7*	CHR9:100174231-100258407
ENSG00000159496	*RGL4*	CHR22:23950638-24059543
ENSG00000139133	*ALG10*	CHR12:34175215-34209675
ENSG00000055332	*EIF2AK2*	CHR2:37311593-37384208
ENSG00000163145	*C1QTNF7*	CHR4:15004297-15447790
ENSG00000171202	*TMEM126A*	CHR11:85359010-85367591
ENSG00000269279	*AL136376.1*	CHR1:116915289-116961197
ENSG00000151743	*AMN1*	CHR12:31800093-31882108

ENSG00000116954	*RRAGC*	CHR1:39303869-39325495
ENSG00000102096	*PIM2*	CHRX:48770458-48776301
ENSG00000159685	*CHCHD6*	CHR3:126423062-126679249
ENSG00000145780	*FEM1C*	CHR5:114856607-114880591
ENSG00000166326	*TRIM44*	CHR11:35684352-35829775
ENSG00000184489	*PTP4A3*	CHR8:142400038-142441620
ENSG00000010818	*HIVEP2*	CHR6:143069579-143266338
ENSG00000161618	*ALDH16A1*	CHR19:49949554-49995565
ENSG00000125347	*IRF1*	CHR5:131817300-131826490
ENSG00000112308	*C6ORF62*	CHR6:24667262-24721064
ENSG00000239839	*DEFA3*	CHR8:6854287-6875823
ENSG00000128708	*HAT1*	CHR2:172640879-172947158
ENSG00000173517	*PEAK1*	CHR15:77400470-77712486
ENSG00000198178	*CLEC4C*	CHR12:7882010-7904201
ENSG00000170296	*GABARAP*	CHR17:7143332-7167302
ENSG00000121236	*TRIM6*	CHR11:5274419-5667019
ENSG00000196262	*PPIA*	CHR7:44836278-44864163
ENSG00000174944	*P2RY14*	CHR3:150803483-151176497
ENSG00000234127	*TRIM26*	CHR6:30152231-30181204
ENSG00000136720	*HS6ST1*	CHR2:128994289-129076151
ENSG00000150756	*FAM173B*	CHR5:10226441-10250027

ENSG00000165949	*IFI27*	CHR14:94571181-94583033
ENSG00000177570	*SAMD12*	CHR8:119201697-119634234
ENSG00000204252	*HLA-DOA*	CHR6:32971954-32977389
ENSG00000102978	*POLR2C*	CHR16:57462080-57521239
ENSG00000146085	*MUT*	CHR6:49398072-49430904
ENSG00000173852	*DPY19L1*	CHR7:34968487-35077883
ENSG00000080802	*CNOT4*	CHR7:135046546-135194875
ENSG00000126822	*PLEKHG3*	CHR14:65170819-65346601
ENSG00000173818	*ENDOV*	CHR17:78234664-78411886
ENSG00000131966	*ACTR10*	CHR14:58466452-58764857
ENSG00000162775	*RBM15*	CHR1:110828996-110889299
ENSG00000119878	*CRIPT*	CHR2:46717888-46852881
ENSG00000164961	*KIAA0196*	CHR8:126036501-126379362
ENSG00000086288	*NME8*	CHR7:37723398-38065297
ENSG00000119616	*FCF1*	CHR14:75179846-75203394
ENSG00000005302	*MSL3*	CHRX:11776277-11793870
ENSG00000116885	*OSCP1*	CHR1:36881427-36916086
ENSG00000110660	*SLC35F2*	CHR11:107661716-107799019
ENSG00000269514	*DKFZP779L1853*	CHR12:48592169-48595814
ENSG00000170633	*RNF34*	CHR12:121837843-122031409
ENSG00000118418	*HMGN3*	CHR6:79910961-79946514

ENSG00000228716	*DHFR*	CHR5:79922046-80172279
ENSG00000156050	*FAM161B*	CHR14:74318546-74551196
ENSG00000143198	*MGST3*	CHR1:165600097-165631033
ENSG00000197608	*ZNF841*	CHR19:52534631-52599018
ENSG00000151914	*DST*	CHR6:56322784-56819426
ENSG00000151882	*CCL28*	CHR5:43376746-43412493
ENSG00000087206	*UIMC1*	CHR5:176332005-176449634
ENSG00000118707	*TGIF2*	CHR20:34894257-35274619
ENSG00000229314	*ORM1*	CHR9:117085335-117088755
ENSG00000110944	*IL23A*	CHR12:56732662-56734193
ENSG00000158109	*TPRG1L*	CHR1:3541565-3546691
ENSG00000100522	*GNPNAT1*	CHR14:53241911-53258386
ENSG00000243414	*TICAM2*	CHR5:114914338-114968689
ENSG00000101236	*RNF24*	CHR20:3912067-3996229
ENSG00000079819	*EPB41L2*	CHR6:131160486-131384462
ENSG00000182010	*RTKN2*	CHR10:63942793-64028466
ENSG00000149489	*ROM1*	CHR11:62369689-62382592
ENSG00000079385	*CEACAM1*	CHR19:42901279-43156507
ENSG00000112304	*ACOT13*	CHR6:24667262-24721064
ENSG00000164136	*IL15*	CHR4:142557751-142655140
ENSG00000131373	*HACL1*	CHR3:15602210-15687329

ENSG00000071127	*WDR1*	CHR4:10075962-10118573
ENSG00000169609	*C15ORF40*	CHR15:83509837-83772195
ENSG00000175643	*RMI2*	CHR16:11343475-11445619
ENSG00000178127	*NDUFV2*	CHR18:9102627-9285206
ENSG00000145779	*TNFAIP8*	CHR5:118604386-118730294
ENSG00000138297	*TIMM23*	CHR10:51592079-51623365
ENSG00000255398	*HCAR3*	CHR12:123011792-123215390
ENSG00000180979	*LRRC57*	CHR15:42783430-42841000
ENSG00000268965	*AC061992.1*	CHR17:76374720-76573476
ENSG00000122786	*CALD1*	CHR7:134429002-134655479
ENSG00000163517	*HDAC11*	CHR3:13518481-13547916
ENSG00000124171	*PARD6B*	CHR20:49348080-49373332
ENSG00000075089	*ACTR6*	CHR12:100550134-100660857
ENSG00000103415	*HMOX2*	CHR16:4511680-4560348
ENSG00000149798	*CDC42EP2*	CHR11:65082288-65089900
ENSG00000080986	*NDC80*	CHR18:2571509-2616634
ENSG00000178741	*COX5A*	CHR15:75212131-75230509
ENSG00000028203	*VEZT*	CHR12:95611521-95696566
ENSG00000173275	*ZNF449*	CHRX:134478720-134497077
ENSG00000157741	*UBN2*	CHR7:138915101-138992981
ENSG00000173917	*HOXB2*	CHR17:46618255-46683776

ENSG00000176124	*DLEU1*	CHR13:50601268-51423190
ENSG00000137996	*RTCA*	CHR1:100731762-100758325
ENSG00000163328	*GPR155*	CHR2:175296965-175351822
ENSG00000215244	*AL137145.1*	CHR10:6319649-6377938
ENSG00000196233	*LCOR*	CHR10:98592016-98740800
ENSG00000166707	*ZCCHC18*	CHRX:103343897-103401708
ENSG00000081177	*EXD2*	CHR14:69649736-69821183
ENSG00000269215	*AC008964.1*	CHR5:39105337-39274630
ENSG00000125954	*CHURC1-FNTB*	CHR14:65381078-65569413
ENSG00000257594	*GALNT4*	CHR12:89813494-89934079
ENSG00000242616	*GNG10*	CHR9:114393631-114432526
ENSG00000153936	*HS2ST1*	CHR1:87380330-87634884
ENSG00000082068	*WDR70*	CHR5:37379313-37753537

Supplementary Table 2 WGCNA coexpressed modules category of lncRNA *RP5-998N21.4*

GENE_SYMBOL	**MODEL**
RP5-998N21.4	BROWN
AKAP3	BROWN
ANKRD22	BROWN
ATP5G3	BROWN
BPI	BROWN
IFIT2	BROWN
CAMP	BROWN
CARD17	BROWN
CCNDBP1	BROWN
CD177	BROWN
CD274	BROWN
IFIT3	BROWN
CEACAM8	BROWN
CRISP3	BROWN
DEFA3	BROWN
DEFA4	BROWN
DST	BROWN
ANXA3	BROWN
FTL	BROWN

GNPNAT1	BROWN
HDAC11	BROWN
HIST1H2AL	BROWN
HIST1H2BB	BROWN
HIST1H3B	BROWN
HIST1H4B	BROWN
HIST1H4D	BROWN
HIST1H4J	BROWN
HIST1H4L	BROWN
GM2A	BROWN
HIST3H2BB	BROWN
HP	BROWN
CDKN2C	BROWN
IL15	BROWN
KRT23	BROWN
LCN2	BROWN
LTF	BROWN
IFI27	BROWN
MMP8	BROWN
MRPS18C	BROWN
MS4A3	BROWN

ORM1	BROWN
OSCP1	BROWN
PLEKHG2	BROWN
PLSCR1	BROWN
ZCCHC18	BROWN
RMI2	BROWN
RNASE2	BROWN
RTP4	BROWN
SELK	BROWN
SLC22A4	BROWN
SNAPC5	BROWN
TCN1	BROWN
TDRD7	BROWN
TGFA	BROWN
TNFRSF4	BROWN
TRIM6	BROWN
VSTM1	BROWN
SEPTIN10	BROWN

Supplementary Table 3 Enrichment of 57 coexpressed humanDEGs in tissue specific expression gene sets.

Tissue	target_tissue_specific_number	target_all_number	coding_tissue_specific_number	coding_all_number	OR	lower	upper	P
Whole.Blood	7	50	145	18068	21.08	7.86	48.36	1.39E-07
Minor.Salivary.Gland	3	50	74	18041	16.10	3.13	51.85	1.13E-03
Spleen	3	50	92	18054	12.84	2.51	41.10	2.11E-03

Supplementary Table 4 Oligo sequences employed in this study.

**Cloning primer s**	**Forward**	**Reverse**
RP5-998N21.4-cDNA	CCCAAGCTTGCGGGGTTGGAGGG ACTCAGCTA	CGGGGTACCTCTTTTTACATTAC ATTTTAATGTGAAT
RBM14-cDNA	ATGAAGATATTCGTGGGCAACGT	CTACATGCGGCGCTGGTAGC
MCM7-cDNA	ATGGCACTGAAGGACTACGCG	TCAGACAAAAGTGATCCGTGTCCG
CTBP1-cDNA	ATGGGCAGCTCGCACTTG	CTACAACTGGTCACTGGCGTG
IFIT2-promoter	GGGGTACCATATTGTGGGGCTTG GGGAAG	CTAGCTAGCCAGGAGGGAACAAA CCACCA
IFIT3-promoter	GGGGTACCCAGGTCTCAAGCCGT TAGGT	CCGCTCGAGTTGCTCTCTGGTCAA TCCCAC
RBM14-shRNA	CCGGCGCGTTTGTTCACATGGAGA ACTCGAGTTCTCCATGTGAACAAA CGCGTTTTTG	AATTCAAAAACGCGTTTGTTCACA TGGAGAACTCGAGTTCTCCATGTG AACAAACGCG

**RT-qPCR primer s**	**Forward**	**Reverse**
RP5-998N21.4	CTCTCAGTCTCACAGTCCCTTAG	CCCTTGTCATATTCTGTTGGCTAG A
IFIT2	CACTGCAACCATGAGTGAGAAC	AGGCCAGTAGGTTGCACATT
IFIT3	GAAACAGCCATCATGAGTGAGG	GGCTGCCTCGTTGTTACCAT
ANXA3	TTAGCCCATCAGTGGATGCTG	CTGTGCATTTGACCTCTCAGT
MCM7	CGCGCTAGAGAAGGAAAAGG	CGGCATCAGCAAAGAGCTTC
CTBP1	GGCCTGCCGCTTGTAAGACT	GTCTCGAGCCAAAGTGCTCA
RBM14	ACTTGGAAGATTTTCGTGGGC	TCCTTCTCCATGTGAACAAACG
CXCL10	GCTTCCAAGGATGGACCACA	GCAGGGTCAGAACATCCACT
GAPDH	AATGGGCAGCCGTTAGGAAA	GCCCAATACGACCAAATCAGAG

**ChIP-qPCR primer s**	**Forward**	**Reverse**
IFIT3-1	AGAGGGATCTTGATAGGGTTCCA	GCTGAAGAGGTTTCCACCCA
IFIT3-2	GCCCTACTCTCCCACCCCTTTA	ACTGACCTCATGACTGCCCT
IFIT2-1	CTCCAGCAGGAAACCAGTGTA	CCTGAGCTGATGGCAGATTCA
IFIT2-2	ACGTCAGCTGAAGGGAAACAA	GCACTACCGAATACGAAGGGA
ANXA3	TTCGCAGTTTACTCGCACAC	TCTGAAATGCAGGACTGAGG

**ChIRP-qPCR primers**	**Forward**	**Reverse**
IFIT3	TCATTTTCCTCCTCCCAACGAT	TGGTGACCAGGCTGATTTGTT
IFIT2-1	CAGGCCGATGAAACATCCCT	TACAAGTGGCCTCTGGTTCC
IFIT2-2	TTAAGTGAAGCACCAGGCCG	TTTCCTCCGGAGCTGAGTTG
SNPRNA70	GGGAGATACCATGATCACGAAGGT	CCACAAATTATGCAGTCGAGTTTCCC
GAPDH	ATGTTGCAACCGGGAAGGAA	AGGAAAAGCATCACCCGGAG

**ChIRP Probe**
/5bio/-TGGTTATGTGTAGAGGTGTT
/5bio/-TCTGATGGAGGTATGTTACA
/5bio/-GGAATGATTGTTGAGTAGGA
/5bio/-TTTAGACATTTCCAGCACCT
/5bio/-ATTTTCCCATTCTGCTGAGG
/5bio/-GACCCGAGCCCTAATCATAT
/5bio/-ACTGACTTCCAGCACCATAG
/5bio/-GAATTCAATTGCAGCTCACT
/5bio/-GGAATAAGGGAGGGAAGAGA
/5bio/-CCCAGTGAGAAATTATTTGC

SUPPLEMENTARY DATA

Supplementary Data 1. GO biological pathway analysis of 57 coexpressed humanDEGs.

Supplementary Data 2. DEGs identified from the lncRNA *RP5-998N21.4* overexpressed SK-N-SH cells.

Supplementary Data 3. GO-BP analysis of 1722 SK-N-SH cells DEGs.

Supplementary Data 4. Correlation of *RP5-998N21.4* with IFIT2, IFIT2 and ANXA3 in GTEx.

Supplementary Data 5. 142 proteins that were predicted to significantly interact with *RP5-998N21.4* by LncADeep.

Supplementary Data 6. 646 proteins that bound significantly to *RP5-998N21.4* by mass spectrometry analysis.
