# Supplementary material for: LncRNA RP5-998N21.4 promotes immune defense through upregulation of IFIT2 and IFIT3 in schizophrenia
Source: Schizophrenia (Heidelb). 2022 Mar 1;8(1):11. doi: 10.1038/s41537-021-00195-8 (PMC8888552; doi:10.1038/s41537-021-00195-8)
Supplement: Supplementary file 8 — Reporting Summary [file 41537_2021_195_MOESM8_ESM.pdf]

nature portfolio

Corresponding author(s): Cunyou Zhao

Last updated by author(s): Nov 2, 2021

Reporting Summary

Nature Portfolio wishes to improve the reproducibility of the work that we publish. This form provides structure for consistency and transparency in reporting. For further information on Nature Portfolio policies, see our Editorial Policies and the Editorial Policy Checklist.

Statistics

For all statistical analyses, confirm that the following items are present in the figure legend, table legend, main text, or Methods section.

n/a	Confirmed	
☐	☒	The exact sample size (*n*) for each experimental group/condition, given as a discrete number and unit of measurement
☐	☒	A statement on whether measurements were taken from distinct samples or whether the same sample was measured repeatedly
☐	☒	The statistical test(s) used AND whether they are one- or two-sided<br>*Only common tests should be described solely by name; describe more complex techniques in the Methods section.*
☐	☒	A description of all covariates tested
☐	☒	A description of any assumptions or corrections, such as tests of normality and adjustment for multiple comparisons
☐	☒	A full description of the statistical parameters including central tendency (e.g. means) or other basic estimates (e.g. regression coefficient) AND variation (e.g. standard deviation) or associated estimates of uncertainty (e.g. confidence intervals)
☐	☒	For null hypothesis testing, the test statistic (e.g. *F*, *t*, *r*) with confidence intervals, effect sizes, degrees of freedom and *P* value noted<br>*Give P values as exact values whenever suitable.*
☒	☐	For Bayesian analysis, information on the choice of priors and Markov chain Monte Carlo settings
☒	☐	For hierarchical and complex designs, identification of the appropriate level for tests and full reporting of outcomes
☐	☒	Estimates of effect sizes (e.g. Cohen's *d*, Pearson's *r*), indicating how they were calculated

Our web collection on statistics for biologists contains articles on many of the points above.

Software and code

Policy information about availability of computer code

Data collection	All the data related to this manuscript are shown in main text and supplementary files.
Data analysis	There are no custom algorithms or softwares employed in our manuscript.

For manuscripts utilizing custom algorithms or software that are central to the research but not yet described in published literature, software must be made available to editors and reviewers. We strongly encourage code deposition in a community repository (e.g. GitHub). See the Nature Portfolio guidelines for submitting code & software for further information.

Data

Policy information about availability of data

All manuscripts must include a data availability statement. This statement should provide the following information, where applicable:

- Accession codes, unique identifiers, or web links for publicly available datasets
- A description of any restrictions on data availability
- For clinical datasets or third party data, please ensure that the statement adheres to our policy

All data generated in the study are included in the article or uploaded as supplementary materials. RNA-Sequencing data of RP5-998N21.4 overexpressed SK-N-SH cells have been deposited to NCBI GEO site with the GEO number GSE186598. The source data of Figure 3b are provided in Supplementary data 5 and 6. Additional information is also available upon reasonable request to the corresponding authors.

Field-specific reporting

Please select the one below that is the best fit for your research. If you are not sure, read the appropriate sections before making your selection.

☒ Life sciences ☐ Behavioural & social sciences ☐ Ecological, evolutionary & environmental sciences

For a reference copy of the document with all sections, see nature.com/documents/nr-reporting-summary-flat.pdf

Life sciences study design

All studies must disclose on these points even when the disclosure is negative.

Sample size	No clinical analysis was performed in this analyses. No statistical methods were employed predetermine sample size.
Data exclusions	No data were excluded from the analyses.
Replication	Yes
Randomization	No clinical analysis was performed in this analyses.
Blinding	RNA samples were randomly distributed in plates for RNA-sequencing, or qRT-PCR, and all assays were performed blind to diagnosis.

Reporting for specific materials, systems and methods

We require information from authors about some types of materials, experimental systems and methods used in many studies. Here, indicate whether each material, system or method listed is relevant to your study. If you are not sure if a list item applies to your research, read the appropriate section before selecting a response.

Materials & experimental systems

n/a	Involved in the study	
☐	☐	Antibodies
☐	☒	Eukaryotic cell lines
☒	☐	Palaeontology and archaeology
☒	☐	Animals and other organisms
☐	☒	Human research participants
☒	☐	Clinical data
☒	☐	Dual use research of concern

Methods

n/a	Involved in the study	
☒	☐	ChIP-seq
☒	☐	Flow cytometry
☒	☐	MRI-based neuroimaging

Antibodies

Antibodies used	This statement was described in the Methods section.
Validation	The source of antibodies employed in the study were reported in the Methods section.

Eukaryotic cell lines

Policy information about cell lines

Cell line source(s)	SK-N-SH, HEK293T, U251
Authentication	There was no cell lines that were recently authenticated in this study.
Mycoplasma contamination	The cell lines have not been tested for mycoplasma contamination recently.
Commonly misidentified lines (See ICLAC register)	There was no commonly misidentified cell lines used in this study.

Human research participants

Policy information about studies involving human research participants

Population characteristics	This information was described in Human subject analysis subsection of materials and methods section.

Recruitment	This information was described in Human subject analysis subsection of materials and methods section.
Ethics oversight	This information was described in Human subject analysis subsection of materials and methods section.

Note that full information on the approval of the study protocol must also be provided in the manuscript.
